# Identifying Plasma and Urinary Biomarkers of Fermented Food Intake and Their Associations with Cardiometabolic Health in a Dutch Observational Cohort

**DOI:** 10.1021/acs.jafc.2c05669

**Published:** 2023-02-28

**Authors:** Katherine J. Li, Kathryn J. Burton-Pimentel, Elske M. Brouwer-Brolsma, Carola Blaser, René Badertscher, Grégory Pimentel, Reto Portmann, Edith J. M. Feskens, Guy Vergères

**Affiliations:** † Division of Human Nutrition and Health, Department of Agrotechnology and Food Science, Wageningen University & Research, P.O. Box 17, 6700 AA Wageningen, The Netherlands; ‡ Agroscope, Schwarzenburgstrasse 161, CH-3003 Bern, Switzerland

**Keywords:** biomarkers, cardiometabolic health, dietary assessment, fermented foods, metabolomics

## Abstract

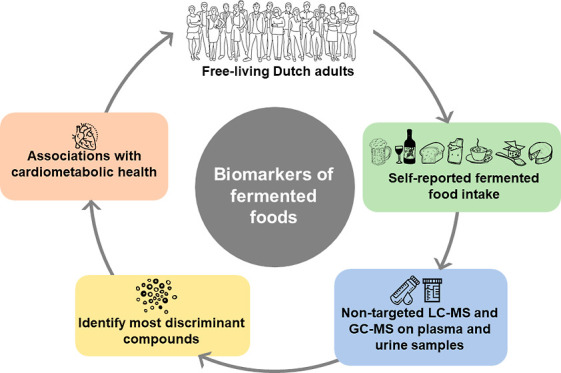

Identification of food intake biomarkers (FIBs) for fermented foods could help improve their dietary assessment and clarify their associations with cardiometabolic health. We aimed to identify novel FIBs for fermented foods in the plasma and urine metabolomes of 246 free-living Dutch adults using nontargeted LC–MS and GC–MS. Furthermore, associations between identified metabolites and several cardiometabolic risk factors were explored. In total, 37 metabolites were identified corresponding to the intakes of coffee, wine, and beer (none were identified for cocoa, bread, cheese, or yoghurt intake). While some of these metabolites appeared to originate from raw food (*e.g.*, niacin and trigonelline for coffee), others overlapped different fermented foods (*e.g.*, 4-hydroxybenzeneacetic acid for both wine and beer). In addition, several fermentation-dependent metabolites were identified (erythritol and citramalate). Associations between these identified metabolites with cardiometabolic parameters were weak and inconclusive. Further evaluation is warranted to confirm their relationships with cardiometabolic disease risk.

## Introduction

Accurate dietary assessment is crucial for detecting potential associations between diet and health. To date, many epidemiological studies still predominantly rely on self-reported dietary assessment methods, such as food frequency questionnaires (FFQ) and 24 h food recalls, which heavily depend on the memory and dedication of the participants.^1,2^ As such, they are prone to multiple sources of measurement errors such as underreporting, inaccurate portion size estimation, and imprecision of food composition databases. Such measurement errors can reduce study power and miss detecting potential associations and may also lead to spurious findings.^3,4^ Additionally, to capture the increasing diversity and complexity of modern diets, self-report methods require extensive food lists, which is burdensome for both participants and researchers. To address these limitations, food intake biomarkers (FIBs) have emerged as a more objective measure of dietary intake. Depending on their specificity, FIBs can be single compounds or a multimarker panel consisting of a combination of different compounds.^5^ Recent advances in nutritional metabolomics have led to the identification of numerous candidate FIBs linked to the ingestion of a food, food group, or a dietary pattern.^3,6^ However, FIBs for many foods in the diet have yet to be explored and validated—including fermented foods.

Fermented foods have been consumed since the beginning of human civilization and comprise up to 40% of the human diet.^7,8^ The fermentation process not only improves the shelf life and organoleptic qualities of food, but it can also impart novel nutritional qualities that could improve human health.^9,10^ A number of dietary intervention and epidemiological studies have suggested that the consumption of fermented foods positively affects cardiometabolic health, including weight maintenance, glucose metabolism, and cardiovascular health,^9,11−14^ but the evidence is inconclusive. Thus, identification and validation of FIBs for fermented foods could improve the accuracy of dietary assessment and support further studies in obtaining more conclusive diet–health associations. Additionally, FIBs could also help elucidate the mechanisms of action that underpin the purported health benefits of fermented foods.

We previously conducted a systematic review of FIBs of fermented foods consumed worldwide and found several candidate FIBs at the food level, food group level, and/or fermentation level for several fermented foods, including wine, beer, bread, cocoa, coffee, postfermented tea, fermented soy, cheese, and yoghurt.^15^ The majority of these FIBs were identified in postprandial studies with a small number of participants, and their relevance needs to be explored in free-living populations with complex, uncontrolled diets.^16^ In the current work, we aimed to identify further FIBs of fermented foods consumed in The Netherlands by analyzing the plasma and urine metabolomes of a Dutch adult cohort using LC–MS and GC–MS. By utilizing a larger, free-living population, we expected the FIBs that emerge would be considered to be the most powerful and reliable indicators of habitual fermented food intake. In addition, we examined associations between the identified FIBs and several cardiometabolic risk parameters and composite risk scores.

## Materials and Methods

### Study Population

The Nutrition Questionnaires plus (NQplus) study is a prospective cohort study of 2048 Dutch men and women (20 to 70 years) with the aim to gather extensive data on participant demographics, anthropometrics, lifestyle, medical history, and cardiometabolic health outcomes.^17,18^ Participants were recruited between June 2011 and February 2013. All measurements were performed according to a standardized protocol by trained research personnel. The study was approved by the ethical committee of Wageningen University and Research (protocol number NL34775.081.10) and conducted in agreement with the Declaration of Helsinki. Written informed consent was obtained from all participants prior to the start of the study.

Metabolomics analyses were performed on a subcohort of NQplus participants (*n* = 531; *n* = 485 with plasma samples and *n* = 492 with urine samples) (herein referred to as the “metabolomics subcohort”). These participants were initially selected based on having a biosample collected within 14 days of completing either a FFQ or a 24 h recall. The FFQ was preferred over the 24 h recalls since it reflects more precisely the intake on any given day and is less sensitive to fluctuations in daily intake. Thus, for the selection of the most discriminant metabolites for identification, we focused the analyses on *n* = 246 unique participants who had a biosample collected within 14 days of completing a FFQ (*n* = 228 with plasma samples, and *n* = 216 with urine samples) (herein referred to as the “identification subcohort”, which is contained within the “metabolomics subcohort”). This criterion ensured that biosample collection occurred within the FFQ reference period of 1 month. To explore the stability of the FIBs with increasing time between biosample collection and FFQ completion, additional correlation analyses were conducted among participants with biosample collection within ±30 days (*n* = 273), ±90 days (*n* = 354), and ±180 days (*n* = 501) of completing the FFQ, as well as within all 531 participants in the metabolomics subcohort.

### Food Frequency Questionnaire and Levels of Fermented Food Intake

A detailed description of the validated, self-administered, semiquantitative 216-item FFQ used to assess habitual dietary intake has been reported previously.^17,18^ In, participants completed the FFQ online and answered questions relating to frequency by selecting 1 of 10 frequency categories ranging from “never” to “6–7 days per week”. Portion sizes were estimated using commonly used household measures. Total food intake (in g/d) was determined by multiplying consumption frequency by portion size as defined in the Dutch food composition tables (2011).^19^ A total of 39 food items were classified as fermented, using criteria described previously^8^ (Table S1). Most of the fermented foods and food groups in the FFQ have already been judged to have a good agreement with the intakes reported in 24 h recalls.^8^ Only fermented foods and food groups that achieved “adequate” to “good” agreement in the validation study^8^ (which is important for determining the reliability of self-reported intakes) and were consumed by at least a third of the population (which is important for the detection of potential FIBs in biosamples and selection of the most relevant FIBs) were included in the current analyses. These included fermented beverages (coffee, beer, and wine), fermented cereals/grains (white bread and whole-grain bread), fermented dairy (cheese and yoghurt), and cocoa-based products.

To facilitate the selection of FIBs that reflect the absolute dry weight of the different fermented foods considered within the fermented food groups (beverages, cereals/grains, cocoa-based products, and dairy), we further calculated the g dry matter/day intakes for each fermented food by subtracting the water weight of each food (in g/day) from the total intake (in g/day) (water weight determined from the Dutch food composition tables). Subsequently, energy adjustment was performed on all individual fermented foods as well as fermented food groups using the commonly used residual method.^20^ All energy-adjusted fermented food intakes (in g/day and g dry matter/day) were then divided into tertiles representing the low (T1), mid (T2), and high (T3) levels of intake.

### Cardiometabolic Health Parameters

Ten cardiometabolic health parameters collected at the baseline were included in the current analysis.^18^ Height was determined using a stadiometer (SECA, Germany, nearest 0.1 cm), and weight was determined using a digital weighing scale (SECA, nearest 0.1 kg). The BMI was calculated by dividing weight (in kg) by height (in m^2^). Waist circumference was measured twice using a nonflexible measuring tape (SECA 201, nearest 0.5 cm) and averaged. Enzymatic methods^21^ were applied to assess fasting plasma glucose, total cholesterol, HDL cholesterol, and serum triglycerides using a Dimension Vista 1500 automated analyzer (Siemens, Erlangen, Germany) or Roche Modular P800 chemistry analyzer (Roche Diagnostics, Indianapolis, USA). Plasma LDL cholesterol was calculated with the Friedewald equation.^22^ Hemoglobin A1c (HbA1c) concentrations in whole blood were determined by HPLC using an ADAMS A1c HA-8160 analyzer (A. Menarini Diagnostics). Systolic and diastolic blood pressure were measured using a digital blood pressure monitor (IntelliSense HEM-907, Omron Healthcare, USA); the first measurement was discarded, and the remaining (up to 6) measurements were averaged. Participants were classified as having hypertension, suboptimal cholesterol, or type II diabetes based on the cutoffs and definitions described in relevant guidelines of the European Society of Cardiology/European Atherosclerosis Society (ESC/EAS)^23−25^ and having metabolic syndrome based on the harmonized guidelines of the International Diabetes Federation (IDF) et al.^26^

Two composite risk scores were also determined as previously described,^27^ consisting of a continuous metabolic syndrome (MetS) score using summed age- and sex-adjusted standardized residuals (*z*-scores) of individual MetS parameters^28−30^ and the European Systematic COronary Risk Evaluation (SCORE)^31,32^ evaluating 10 year risk of fatal cardiovascular disease.

### Covariates

All covariate data relevant to the current work (age, sex, education level, smoking status, physical activity, alcohol consumption, and dietary intake) were collected via questionnaires.^18^ For educational level, participants with no education or primary/lower vocational education were classified under “low”, participants who completed lower secondary or intermediate vocational education were classified under “intermediate”, and participants who completed higher secondary or higher vocational education, or university, were classified under “high”. A “smoker” was defined as a current smoker and former smoker who quit >35 years old and a “nonsmoker” as never smoker and former smoker who quit <35 years old.^33^ Information on the participants’ usual physical activity over the past 4 weeks was obtained using the validated Activity Questionnaire for Adults and Adolescents (AQuAA), which provides the time spent on sedentary-, light-, moderate-, and vigorous-intensity activities in min/week.^34^ Intake levels of alcohol and different foods were assessed by a FFQ, as described above.

### LC–MS Metabolomics Analysis

EDTA plasma and 24 h urine samples collected for NQplus were used for metabolomics analyses. All samples were thawed on ice and kept at 4 °C during analysis. Prior to LC–MS analysis, phospholipids were removed from plasma samples to limit ion suppression using a Phree filter (Phenomenex Inc., Torrance, CA). Urine samples were normalized based on the specific gravity as determined by the refractive index (refractometer RE40, Mettler Toledo, Switzerland), as described previously.^35,36^ LC–MS metabolomics analysis was performed using an UltiMate 3000 RS UPLC system (Thermo Fisher Scientific, Waltham, MA) with a Waters Acquity UPLC HSS T3 column (length 150 mm, diameter 2.1 mm, and particle size 1.8 μm), coupled with a maXis 4G+ quadrupole time-of-flight mass spectrometer (Bruker Daltonik GmbH, Bremen, Germany). A gradient was run from 5% to 95% of mobile phase A within 15 min at 0.4 mL/min. Mobile phase A consisted of Milli-Q water with 0.1% formic acid, and mobile phase B consisted of acetonitrile with 0.1% formic acid. The column was heated to 35 °C with a postcolumn cooler set to 25 °C. The resulting system pressure was ∼600 bar, dependent on the actual composition of the mobile phase at the specific time. The mass spectrometer ESI was operated in positive ion mode, and spectra were recorded from 75 to 1500 *m*/*z*. Collision-induced dissociation was performed using energies from 20 to 70 eV. 5 μL of filtered plasma or normalized urine from each sample was injected once in a randomized sequence. Quality control (QC) pools were prepared from plasma or urine samples by mixing all samples of each sample type at equal volumes. QC samples were injected at five sample intervals for signal drift correction. Blanks (consisting of ultrafiltered LC–MS-grade water) were also injected at the beginning and end of each batch for the detection of contaminants.

Progenesis QI (v.2.3.6198.24128, NonLinear Dynamics Ltd., Newcastle upon Tyne, United Kingdom) was used for retention time correction, peak-picking, deconvolution, adducts annotation, and normalization (default automatic sensitivity and without minimum peak width). All solvents and reagents were purchased from Sigma-Aldrich Chemie GmbH (Buchs, Switzerland).

### GC–MS Metabolomics Analysis

Plasma and urine samples were prepared for GC–MS analysis as described previously for serum^37^ and urine.^38^ Specifically, for each 100 μL plasma sample, 50 μL of an internal standard solution (labeled d-sucrose, 13C12, 98%, Cambridge Isotope Laboratories, Inc., Cambridge, UK, *c* ≈ 0.16 mg/mL in water) was added, followed by precipitation with 300 μL of cold methanol, centrifugation, transfer of supernatant (370 μL), and drying using a vacuum centrifuge. Urine samples were normalized prior to analysis using the refractive index methods described above for the LC–MS analysis. For each 100 μL urine sample, 50 μL of an internal standard solution (labeled d-sucrose) was added and dried using a vacuum centrifuge. The plasma and urine samples further underwent a two-step derivatization (methoximation with *O*-methylhydroxylamine hydrochloride followed by silylation with *N*-methyl-*N*-(trimethylsilyl)trifluoroacetamide (MSTFA)) and were analyzed by GC–MS 7890B/MS5977A (Agilent Technologies, Santa Clara, CA, US) with a CombiPAL autosampler (CTC-Analytics AG, Zwingen, Switzerland) and a DB-5 ms fused silica capillary column (60 m, 0.25 mm i.d., 0.25 μm film thickness, Agilent Technologies, Basel, Switzerland). The samples were injected using a multimode injector according to the following temperature program: initially 90 °C, a heating rate of 900 °C/min until 280 °C, held for 5 min and cooled at a rate of −30 °C/min, and maintained at 250 °C. The oven program was as follows: initial temperature 70 °C for 2 min, increase up to 160 °C at a rate of 5 °C/min, increase to 300 °C at a rate of 10 °C/min, which was held for 36 min, equilibration time 1 min. The MS detection mass ranged from 28.5 to 600 Da, the MS source temperature was 230 °C, and the MS Quad temperature was 150 °C. Electron ionization was performed with 70 eV. Each batch was initiated by three injections of QC samples for equilibration and after every fifth plasma sample, a fresh QC was injected. At the start and end of each batch, a blank sample (Milli-Q water) was included. QC samples and blank samples underwent the same sample preparation as plasma samples.

Agilent data files acquired from GC–MS analysis were deconvoluted and converted into CEF files using Agilent MasshunterProfinder (Agilent Technologies, Santa Clara, US). Data files were further processed in Agilent Mass Profiler Professional (Agilent Technologies, Santa Clara, U.S.) to perform alignment and compound identification. Features with retention time before 10 min (reagents region) were removed. All markers selected based on deconvoluted data were further evaluated using a targeted approach in order to optimize integration. Using RI, quantifier and qualifier ion retrieved from deconvoluted data, the suggested markers were analyzed in MassHunter Quantitative Analysis (Agilent Technologies, Santa Clara, US). The peak integration was checked in each sample individually. Responses from the quantifier ion of marker compounds were normalized with the response of the quantifier ion of internal standard [labeled d-sucrose (ion 220)].

### Metabolomics Data Preprocessing

**Figure 1:**
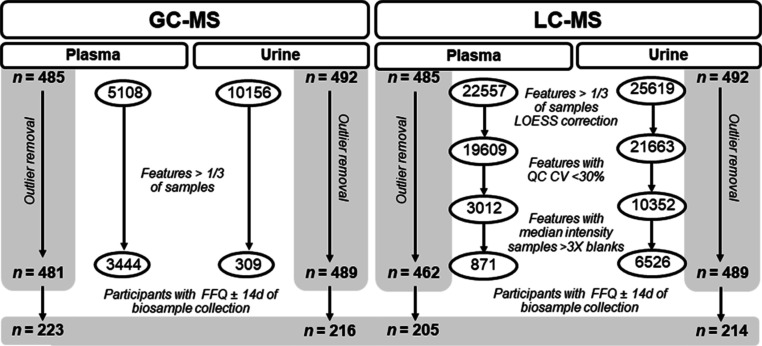
GC–MS and LC–MS metabolomics workflow for feature filtration and outlier removal.

The dataset was corrected to account for signal drift and reduced via multiple filtering steps to remove features with poor repeatability and potential contaminants (Figure 1). Principal component analyses (PCAs) of the QCs for both LC–MS and GC–MS present the relative stability of the analysis (Figure S1). For LC–MS, the QC-based robust locally estimated scatterplot smoothing signal correction method was applied for signal drift correction^39^ using R (v.3.6.3).^40^ Features resulting from LC–MS analysis were removed if they had poor repeatability (detected in less than one-third of samples), a relative standard deviation > 30% in the QC samples, and a median in the QC samples that was <3 times higher than the median calculated for the blanks. For GC–MS, features detected in less than one-third of samples were removed (features that had high levels in blanks or originated from the GC column were removed after identification to ensure all features captured during automatic detection are retained and further inspected for relevance). Exploratory analyses were performed, and metabolomics sample outliers, defined as observations clearly falling outside Hotelling’s T2 tolerance eclipse (95% confidence interval) in the PCA score plot, were identified and excluded (*n* = 23 LC–MS plasma, *n* = 3 LC–MS urine, and *n* = 4 GC–MS plasma, *n* = 3 GC–MS urine) (Figure S2).

### Selection of Discriminant Metabolites by Univariate and Multivariate Statistics

We performed several complementary univariate and multivariate statistical tests to select and confirm the most consistent features to proceed with metabolite identification.^41^ Differences in levels of features by tertiles of intake for fermented foods and groups (T1, T2, and T3) were assessed by a Kruskal–Wallis test followed by a posthoc Conover-Iman pairwise comparison test. An additional step was conducted to select features with higher levels in higher tertiles compared to lower tertiles (*i.e.*, a median of T3 > T1, T3 > T2, and T2 > T1). To determine the strength and direction of the associations between fermented food intakes and features, nonparameteric Spearman’s rank correlation coefficients (*r*_s_) were calculated; significant correlations with *r*_s_ > 0.20 were selected for further analysis. For all univariate statistical tests, *p*-values were adjusted for the false discovery rate (FDR) using the method of Benjamini and Hochberg,^42^ and FDR-adjusted *p* ≤ 0.05 was set as the significance threshold.

Two multivariate tests were also conducted to further unveil and confirm features that discriminate between tertiles of fermented food intake. Partial least-square discriminant analysis (PLS-DA) was performed to identify features that differentiate the lowest and highest tertiles of intake for each fermented food or food group (SIMCA-P software v.15.0; Umetrics). The dataset was scaled using the unit variance (UV) method. The quality and validity of the models were evaluated by the goodness-of-fit parameter (R2Y > 0.5), the predictive ability parameter (Q2 > 0.2), and permutation tests with 999 random permutations to exclude any random separation of the sample groups.^43^ Permutation plots (correlations of the observed and permuted data on the *X*-axis against the R2Y and Q2 on the *Y*-axis) for all models were visually interpreted as follows: the model was considered to be well guarded against overfitting if the Q2 values of the permuted dataset were lower than the Q2 value of the actual dataset to the observed dataset. Finally, the most discriminant features from these models were selected based on variable importance in projection (VIP) scores (VIP > 1 as a cutoff value). Second, we used random forests to model these data and further select the most discriminant features between T1, T2, and T3 of fermented food or food group intake, using the randomForest package.^44^ The dataset was split into training (0.75) and test (0.25) datasets. For tuning the random forest, the number of trees ranged from 500 to 800 and the node sizes from 1 to 10. The “mtry” parameter was set to *x* (0.01, 0.05, 0.15, 0.25, 0.333, and 0.4), where *x* is the number of features considered for the model. We then implemented a full Cartesian grid search to choose the best model using the out-of-bag estimates generated from the random forest model. The results and variable importance from this step were further subjected to permutation testing using the “altmann” method (*n* = 500)^45^ applied in the ranger package.^46^ For features selected from multivariate analysis, the Wilcoxon test (for two comparisons) or Kruskal–Wallis test followed by a posthoc Conover-Iman pairwise comparison test (for three comparisons) was also conducted (non-FDR-adjusted *p* ≤ 0.05) as a separate validation test of the features selected from these models.

Given the large number of significant features revealed across complementary univariate and multivariate tests (586 plasma and 151 urinary metabolites from GC–MS and 110 plasma and 4473 urinary metabolites from LC–MS; data not shown in tables), those significant in at least two of the four statistical tests were prioritized and selected for identification. For urinary features measured by LC–MS, a large number of features remained significant; thus, an additional criterion of Spearman’s FDR *p*-value ≤ 1 × 10^–10^ had to be applied to select a number of features that could feasibly be identified. A summary of the significant features across at least two of the four statistical tests (and prioritized for identification) are provided in Table S2. Aside from PLS-DA, all analyses were performed in R (v.3.6.3).^40^

### Metabolite Identification

For LC–MS, the Human Metabolome Database,^47^ the MassBank of North America,^48^ the National Institute of Standards and Technology database (NIST v17), and METLIN^49^ were used to screen the identity of metabolites with a 10 ppm mass accuracy threshold. Identity suggestions from databases were then screened based on the chemical and biological relevance of each suggested metabolite identification (as provided on HMDB and/or through a search of the compound name on PubMed and Google) and confirmed by MS fragmentation data (where available). Pure analytical standards were then purchased for the tentatively identified and most biologically plausible compounds and injected at two concentrations in sample QCs and in the solvent. For GC–MS, the Golm Metabolome Database^50^ and NIST v17 were used to screen the identity of compounds, and an internal database of internal standards was used to confirm the metabolite identification. In the case that stereoisomeric forms of selected discriminating features were identified, the peak with a higher response was further evaluated. The list of standard suppliers is provided in Table S3. For both LC–MS and GC–MS, the level of identification of each discriminant metabolite is defined according to the Metabolomics Standards Initiative (MSI) recommendations,^51^ as follows: level 1, compounds identified by comparison to a pure reference standard based on spectral data (LC: molecular weight with a 10 ppm accuracy threshold, fragmentation pattern when available, isotopic distribution, and retention time with a 10% accuracy threshold; GC: based on spectral data and retention indices (RIs) with a 5% accuracy threshold and 10% for very large peaks); level 2, based on spectral data but without chemical standards (LC: fragmentation pattern match to library spectral data of at least two major peaks; GC: library match factor >80%); level 3, putatively characterized compound classes; and level 4, unknown compound. Details of the identification features of metabolites analyzed from GC–MS (37 plasma and 75 urinary metabolites) and LC–MS (13 plasma and 89 urinary metabolites) are presented in Tables S4 and S5, respectively. The metabolites corresponded to the intakes of total fermented beverages (FBs) (number of metabolites: 112), wine (89), coffee (72), beer (17), white bread (9), total fermented cereals/grains (FCG) (1), total fermented dairy (FD) (1), cheese (1), and cocoa (1) (none for whole-grain bread or yoghurt).

### Associations between Identified Metabolites and CMD Risk Parameters

Participant characteristics are reported as number (percentages), mean (standard deviation) for normally distributed variables, or medians (interquartile range) for skewed variables. Multivariable adjusted linear regression and restricted cubic spline regression were used to evaluate the associations between the identified metabolites and CMD risk factors. CMD risk parameters acting as dependent variables that were not normally distributed were log-transformed, which included: BMI, plasma HbA1c, plasma glucose, serum triglycerides, and SCORE. All variables were normalized by *z*-scores prior to analysis to allow comparability across associations. Analyses were performed unadjusted (model 0), adjusted for age (years) and sex (male, female) (model 1) + physical activity (minutes/week), smoking (smoker/non-smoker), and education level (high, intermediate, low) (model 2) + dietary factors (g/day) (model 3). For associations with continuous MetS, which already takes into account age and sex, analyses were performed unadjusted (model 0) and fully adjusted for smoking, physical activity, education, and dietary factors (model 3). For associations with SCORE, which already takes into account age, sex, and smoking status, analyses were performed unadjusted (model 0) and fully adjusted for physical activity, education, and dietary factors (model 3). Dietary factors included in the fully adjusted models included those indicated in the literature to be important for CMD risk in addition to those significantly correlated with the identified metabolites and included vegetables, fruits, alcohol, meat, and confectionary/desserts. All analyses were performed in R (Version 3.6.3).^52^ For all models, the level of significance was set at *p* ≤ 0.05. To account for multiple comparisons, FDR-adjusted *p*-values are also presented.

## Results

### Characteristics of the Population

The characteristics of the participants in the metabolomics and identification subcohorts are presented in Table 1. The median age of the participants was ∼58 years, and the majority were highly educated (>60%) and nonsmokers (>69%). No significant differences were observed in background demographics between the two subcohorts. Among the dietary factors, participants in the identification subcohort had significantly higher intakes of total energy, fat, sodium, beer, soft drinks, and egg products compared to participants in the metabolomics subcohort but with a similar interquartile range (significant differences were also observed for tea intake but medians were comparable) (*p* ≤ 0.05). Among cardiometabolic parameters, participants in the identification subcohort have a slightly larger waist circumference, higher systolic blood pressure, and lower plasma HDL-cholesterol than participants in the metabolomics subcohort. However, although significant, the differences observed are relatively minor and do not pertain to the broader indicators of health linked to each measure (*e.g.*, BMI, hypertension, and suboptimal cholesterol). The distribution of participant risk for continuous MetS and SCORE is presented in Figure S3.

**Table 1:** Characteristics of the Study Population^a^

characteristic	metabolomics subcohort (*n* = 531)	identification subcohort (*n* = 246)	*p*-value
Demographics			
age, years	57 (46–63)	58 (46–65)	0.26
education, *n* (%)			0.73
low	37 (7)	19 (8)	
intermediate	148 (28)	77 (31)	
high	344 (65)	149 (61)	
smoking status, *n* (%)			0.20
smoker	118 (26)	70 (31)	
nonsmoker	343 (74)	159 (69)	
physical activity, min/week	2136 ± 1093	2043 ± 1046	0.37
supplement use, *n* (%)	0.8 ± 1.2	0.7 ± 1.2	0.58
Dietary Factors			
total energy intake, kcal/day	2128 ± 499	2220 ± 530	0.02*
Macronutrients			
fat, g/day (En %)	84 ± 25 (36%)	90 ± 27 (36%)	0.01*
carbohydrates, g/day (En %)	230 ± 60 (43%)	237 ± 63 (43%)	0.17
protein, g/day (En %)	77 ± 18 (14%)	80 ± 18 (14%)	0.06
fiber, g/day	25 ± 7	25 ± 7	0.80
sodium, mg/day	2261 ± 653	2375 ± 711	0.03*
Fermented Foods and Groups			
total fermented beverages, g/day	592 (324–799)	629 (406–865)	0.26
coffee, g/day	406 (174–638)	406 (196–638)	0.48
wine, g/day	25 (4–87)	20 (0–80)	0.31
beer, g/day	9 (0–79)	20 (0–118)	0.04*
total fermented cereals/grains, g/day	130 (88–166)	133 (88–170)	0.41
whole-grain bread, g/day	80 (47–112)	77 (41–114)	0.51
white bread, g/day	2 (0–8)	2 (0–10)	0.24
cocoa, g/day	4 (1–8)	4 (1–8)	0.80
total fermented dairy, g/day	152 (76–245)	151 (69–240)	0.79
cheese, g/day	25 (13–42)	28 (14–46)	0.24
yoghurt, g/day	89 (29–139)	82 (21–139)	0.30
Other Foods and Groups			
tea, g/day	174 (67–406)	174 (67–406)	0.04*
alcoholic drinks, g/day	81 (18–207)	108 (19–245)	0.26
soft drinks, g/day	5 (0–42)	13 (0–54)	0.04*
fruits, g/day	217 (86–238)	166 (81–233)	0.10
vegetables, g/day	150 (97–204)	140 (94–196)	0.11
potatoes, g/day	67 (37–87)	67 (37–87)	0.29
legumes, g/day	38 (19–79)	38 (22–79)	0.89
meat products, g/day	72 (46–98)	79 (54–105)	0.053
eggs and egg products, g/day	9 (7–18)	14 (7–18)	0.03*
fish, g/day	11 (6–16)	11 (6–16)	0.72
nuts and seeds, g/day	13 (6–25)	13 (6–26)	0.71
sauces, spreads, and cooking fats, g/day	41 (28–54)	42 (30–57)	0.21
salty and processed snack foods, g/day	35 (16–59)	37 (20–64)	0.16
sugary confectionary and desserts, g/day	70 (47–104)	78 (50–113)	0.09
Cardiometabolic Factors			
BMI, kg/m^2^	25.1 (22.9–27.2)	25.5 (23.2–28.0)	0.12
BMI category, *n* (%)			0.61
underweight (<18.5 kg/m^2^)	4 (1)	2 (1)	
normal weight (18.5–24.9 kg/m^2^)	249 (7)	103 (42)	
overweight or obese (≥25–29.9 kg/m^2^)	278 (52)	141 (57)	
waist circumference, cm	91 ± 12	93 ± 12	0.04*
diastolic blood pressure, mm Hg	73.7 ± 10.4	74.5 ± 10.8	0.38
systolic blood pressure, mm Hg	125.5 ± 16.0	128.7 ± 16.6	0.01*
hypertension, *n* (%)			0.42
hypertension^b^	109 (20.6)	62 (25.2)	
normal or optimal	421 (79.4)	184 (74.8)	
hypertension treatment, *n* (%)			0.95
being treated with medication and/or diet	69 (13.0)	36 (14.6)	
not being treated	462 (87.0)	210 (85.4)	
plasma total cholesterol, mmol/L	5.4 ± 1.0	5.3 ± 1.0	0.15
plasma LDL cholesterol, mmol/L	3.3 ± 0.9	3.2 ± 0.9	0.63
plasma HDL cholesterol, mmol/L	1.6 ± 0.4	1.5 ± 0.4	0.01*
serum triglycerides, mmol/L	1.0 (0.7–1.4)	1.0 (0.7–1.4)	0.74
suboptimal cholesterol, *n* (%)	398 (75.0)	182 (74.0)	0.84
high cholesterol treatment, *n* (%)			0.76
being treated with medication and/or diet	56 (10.5)	23 (9.3)	
not being treated	475 (89.5)	223 (90.7)	
HbA1c, mmol/mol	35.5 (34.0–38.0)	35.8 (34.0–38.0)	0.97
fasting glucose, mmol/L	5.4 (5.1–5.8)	5.3 (5.0–5.8)	0.11
diabetes, *n* (%)	13 (2.4)	6 (2.4)	0.98
diabetes treatment, *n* (%)			0.77
being treated with medication and/or diet	15 (2.8)	5 (2.0)	
not being treated	516 (97.2)	241 (98.0)	
metabolic syndrome, *n* (%)	67 (12.6)	33 (13.4)	0.85
SCORE, *n* (%)			0.25
≥15%	6 (1.3)	6 (2.6)	
10–14%	17 (3.7)	14 (6.2)	
5–9%	73 (16.0)	42 (18.5)	
1–4%	211 (46.2)	98 (43.2)	
<1%	150 (32.8)	67 (29.5)	

a BMI, body mass index; HDL, high-density lipoprotein; LDL, low-density lipoprotein; SCORE, Systematic COronary Risk Evaluation; SD, standard deviation. Values are presented as mean ± SD unless otherwise specified. Missing values for the metabolomics subcohort: education (*n* = 2), smoking (*n* = 70), physical activity (*n* = 296), LDL cholesterol (*n* = 4), HDL cholesterol (*n* = 4), Hb1Ac (*n* = 5), glucose (*n* = 4), SCORE (*n* = 74). Missing values for the identification subcohort: education (*n* = 1), smoking (*n* = 17), physical activity (*n* = 41), LDL-cholesterol (*n* = 3), HDL-cholesterol (*n* = 3), Hb1Ac (*n* = 4), glucose (*n* = 3), SCORE (*n* = 19). Differences between the metabolomics and identification subcohorts were assessed using the *t*-test (for normally distributed continuous variables), Wilcoxon test (for skewed continuous variables), or χ-squared test (for categorical variables). Significant p-values are bolded and indicated by an asterisk (*).

b Inclusive of grade 1 hypertension, grade 2 hypertension, and isolated systolic hypertension.

### Intake Levels of Different Fermented Foods

The levels of intake of fermented foods in the identification subcohort (mean and tertiles) are presented in Table 2 (in both absolute g/day and g dry matter/day). Out of the fermented food groups evaluated, the highest intake on a g/day basis was total FB followed by total FD, while the highest mean intake of foods on a g dry/matter per day was total FCG. Out of individual fermented foods, coffee had the highest intake among all other fermented foods on a g/day basis (466 g/day) but the lowest intake on a g dry matter/day basis (similar trends were observed for wine and beer). Conversely, intakes of cocoa remained the same regardless of g/day or g dry matter/day (similar trends were observed for white and whole-grain bread, and cheese).

**Table 2:** Tertiles of Fermented Food Intake in the Identification Subcohort (*n* = 246)^a^

	energy-adjusted intakes (g/day)				energy-adjusted intakes (g dry matter/day)			
food group	mean ± SD	T1 (*n* = 82)	T2 (*n* = 82)	T3 (*n* = 82)	mean ± SD	T1 (*n* = 82)	T2 (*n* = 82)	T3 (*n* = 82)
total FB	638 ± 398	264 (124, 378)	615 (551, 679)	978 (857, 1205)	22 ± 20	6 (3, 8)	17 (14, 21)	37 (30, 51)
coffee	466 ± 297	142 (63, 238)	453 (418, 510)	691 (640, 903)	5 ± 3	1 (1, 2)	5 (4, 5)	7 (6, 9)
beer	112 ± 202	–14 (−32, 11)	48 (37, 71)	208 (136, 374)	9 ± 16	–1 (−3, 1)	4 (3, 6)	17 (11, 30)
wine	61 ± 89	3 (−7, 7)	25 (16, 38)	130 (92, 187)	8 ± 12	0 (−1, 1)	4 (2, 5)	17 (12, 27)
total FCG	134 ± 60	79 (61, 94)	132 (115, 144)	182 (168, 219)	85 ± 37	52 (38, 61)	85 (74, 91)	116 (105, 137)
white bread	8 ± 13	–1 (−2, 1)	4 (3, 5)	17 (10, 30)	5 ± 8	0 (−1, 1)	3 (2, 3)	11 (7, 19)
whole-grain bread	82 ± 56	29 (12, 42)	77 (68, 88)	131 (114, 149)	52 ± 35	20 (8, 27)	49 (43, 55)	83 (72, 93)
cocoa	6 ± 9	1 (0, 1)	3 (3, 4)	10 (8, 17)	6 ± 9	1 (0, 1)	3 (2, 4)	10 (8, 17)
tTotal FD	170 ± 121	55 (33, 69)	142 (126, 173)	286 (238, 342)	35 ± 19	16 (11, 21)	32 (28, 38)	54 (47, 63)
cheese	34 ± 26	12 (7, 17)	27 (23, 32)	56 (47, 73)	19 ± 15	7 (4, 10)	16 (13, 18)	31 (27, 42)
yoghurt	89 ± 79	7 (0, 21)	82 (59, 96)	139 (139, 193)	11 ± 10	1 (0, 3)	11 (8, 13)	20 (17, 24)

a FB, fermented beverages; FCG, fermented cereals and grains; FD, fermented dairy. Values are reported as median (IQR) unless otherwise specified.

### Biomarkers Identified for Fermented Food Intake

A total of 12 plasma metabolites and 27 urinary metabolites were identified. An overview of the candidate FIBs identified for various fermented foods and food groups, along with their platforms and biosamples of detection, are presented in Table 3 (plasma) and Table 4 (urine). The majority of the identified metabolites corresponded to the intakes of total FB (7 plasma and 19 urine), which encompasses coffee (3 plasma and 9 urine), wine (3 plasma and 10 urine), and beer (1 plasma and 6 urine). One urinary metabolite identified was discriminant for the intakes of total FCG, and one plasma metabolite was for white bread. However, metabolites discriminant for the intakes of whole-grain bread, cocoa, total FD, cheese, and yoghurt could not be identified.

**Table 3:**
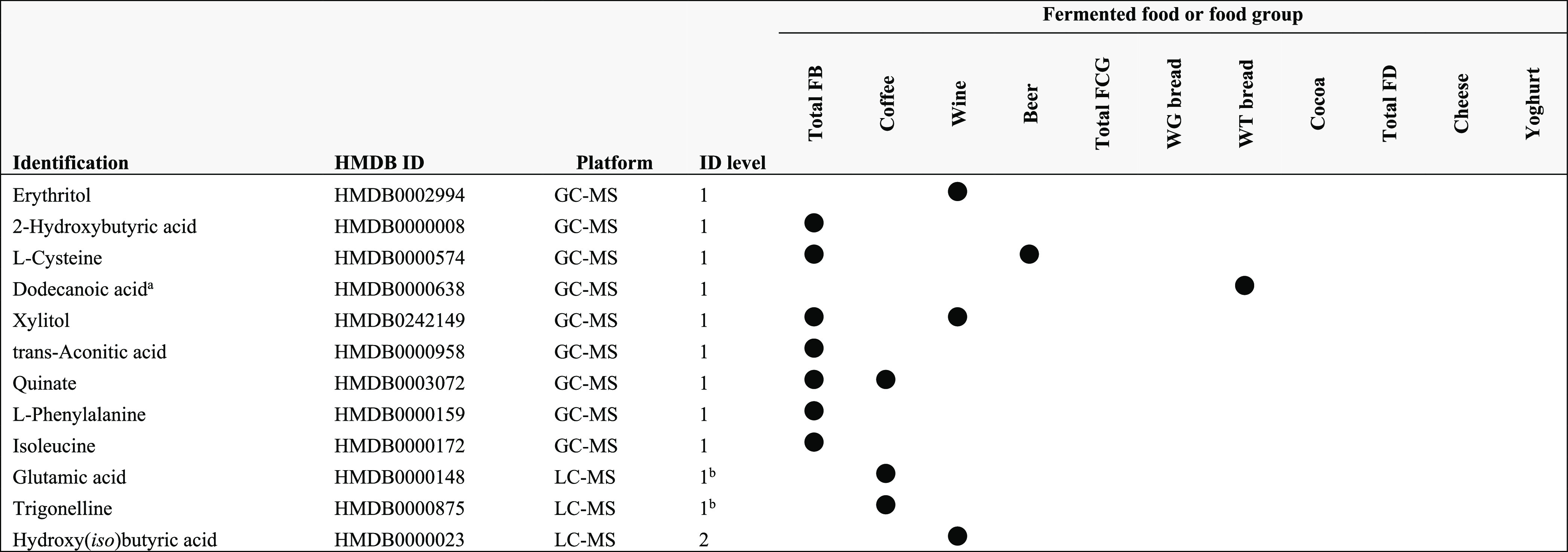
Overview of Identified Plasma Metabolites Discriminant for the Intake of Various Fermented Foods

a FB, fermented beverages; FCG, fermented cereals and grains; FD, fermented dairy; GC–MS, gas chromatography–mass spectrometry; ID, identification; LC–MS, liquid chromatography–mass spectrometry; WG, whole grain; WT, white. Based on Spearman’s correlations this metabolite is negatively associated with the fermented foods and food groups indicated but discriminant based on statistical significance in other tests (*e.g*., PLS-DA, random forest).

b Matched on retention time and mass; information on fragmentation not available.

**Table 4:**
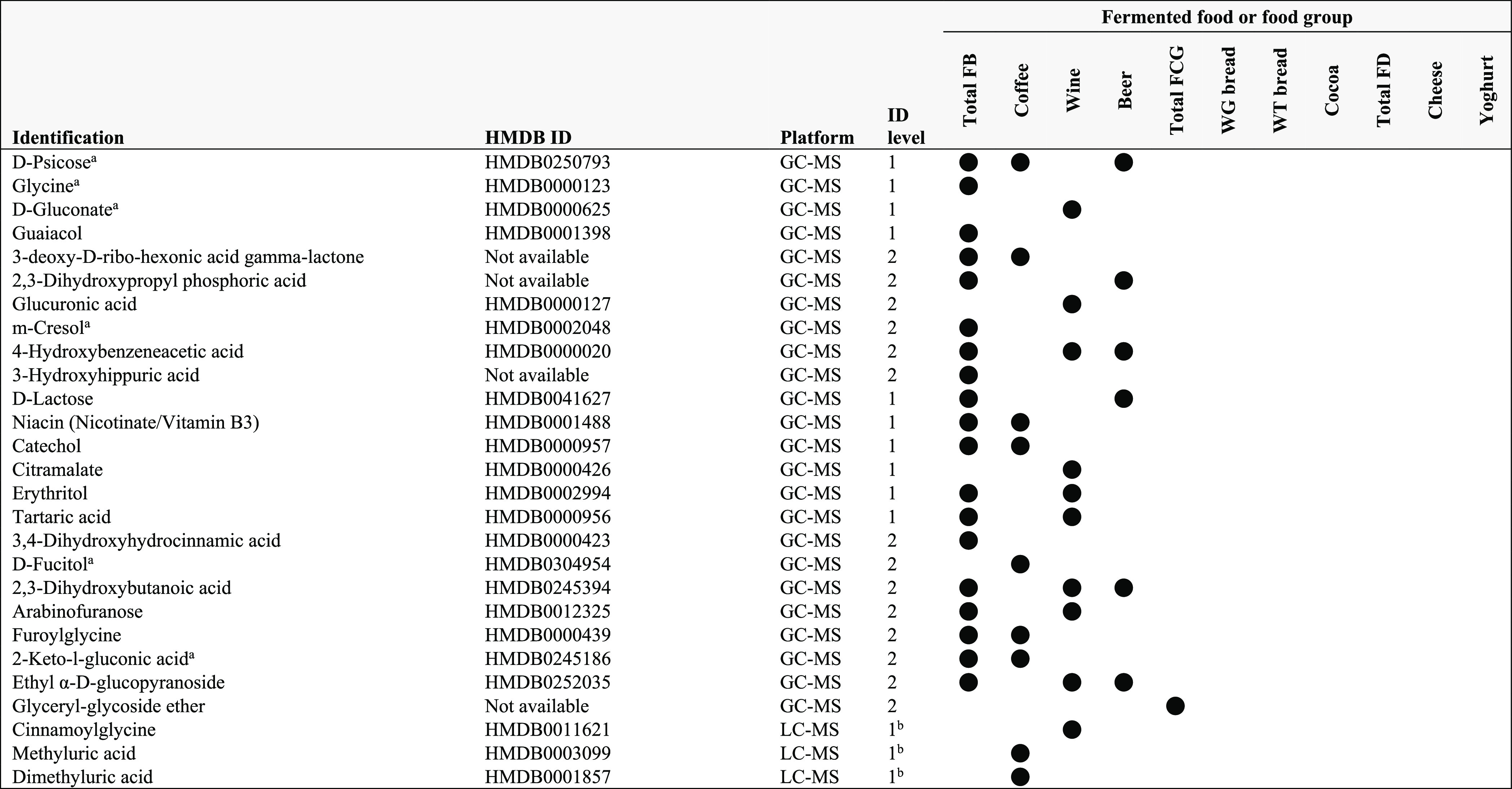
Overview of Identified Urine Metabolites Discriminant for the Intake of Various Fermented Foods

a FB, fermented beverages; FCG, fermented cereals, and grains; FD, fermented dairy; GC–MS, gas chromatography–mass spectrometry; ID, identification; LC–MS, liquid chromatography–mass spectrometry; WG, whole grain; WT, white. Based on Spearman’s correlations this metabolite is negatively associated with the fermented foods and food groups indicated but discriminant based on statistical significance in other tests (*e.g*., PLS-DA and random forest).

b Matched on retention time, mass, and fragmentation pattern.

A closer examination revealed that several of the metabolites (plasma dodecanoic acid, urinary d-psicose, glycine, d-gluconate, *m*-cresol, d-fucitol, and 2-keto-l-gluconic acid) were negatively associated with the fermented foods and food groups indicated (based on Spearman’s correlations, Table S6) but contributed to the discrimination of the intake of these fermented foods based on statistical significance in multivariate tests that do not distinguish between features that are at a higher abundance in a higher tertile intake group (*e.g.*, PLS-DA and random forest). Thus, these metabolites may not be suitable for reflecting fermented food intake and thus are not further discussed as FIBs. However, they may still be important biomarkers in revealing the metabolic effects of consuming these fermented foods.

Several of the identified FIBs overlapped across several fermented foods. Specifically, urinary 2,3-dihydroxybutanoic acid, ethyl α-d-glucopyranoside, and 4-hydroxybenzeacetic acid were discriminant for the intake of total FB, wine, and beer. Several other urinary metabolites also appeared to overlap between two fermented food groups, including 2,3-dihydroxypropyl phosphoric acid and d-lactose (total FB, beer), catechol, furoylglycine, niacin, and 3-deoxy-d-*ribo*-hexonic acid γ-lactone (total FB, coffee), as well as erythritol, tartaric acid, and arabinofuranose (total FB, wine). However, in each case, the significance of the total FB group could be driven by the significance of the individual beverages in this group. Similar overlaps were observed in plasma for l-cysteine (total FB, beer), xylitol (total FB, wine), and quinate (total FB, coffee). One metabolite identified for wine intake (erythritol) was identified in both plasma and urine.

### Stability of the Identified Biomarkers

Spearman’s correlations between the identified FIBs for fermented foods across different times between biosample collection and dietary assessment with the FFQ are presented in Table S6. Almost all correlations observed for the identification cohort (FFQ ± 14 d) remained significant with increasing time between biosample collection and FFQ completion (FFQ ± 30 d, 90 d, 180 d, and all FFQ), with only slight attenuations when the time between biosample collection and FFQ completion increased. The strongest correlations were observed between self-reported coffee intake and a series of FIBs, including plasma quinate, urinary niacin, furoylglycine, methyluric acid, and dimethyluric acid (*r*_s_ ≥ 0.4, *p* ≤ 0.05). For wine, the strongest correlations included urinary tartaric acid and arabinofuranose (*r*_s_ ∼ 0.4), and for beer, the strongest correlation observed was ethyl α-d-glucopyranoside (*r*_s_ ∼ 0.27) (*p* ≤ 0.05). These correlations were also largely echoed between these metabolites and the intake of total FB. For total FCG, a significant moderate correlation was observed between self-reported intake and urinary glyceryl–glycoside ether in the identification cohort (*r*_s_ ∼ 0.37), but the correlation attenuated in the full metabolomics cohort (*r*_s_ < 0.3) (*p* ≤ 0.05). Conversely, correlations for intakes of cocoa, total FD, cheese, yoghurt, white bread, and whole-grain bread and their potential FIBs were either weak or nonexistent.

### Associations between Identified Biomarkers and Cardiometabolic Health Parameters

The results of all associations between the identified biomarkers and CMD risk factors are presented in Table S7. In the fully adjusted model, 21 metabolites were positively associated and 11 were negatively associated with CMD risk parameters (unadjusted *p* ≤ 0.05). After adjusting for multiple comparisons, 11 associations remained significant, including between plasma glutamic acid and urinary 2,3-dihydroxypropyl phosphoric acid with BMI (standardized (Std.) β = 0.28, *R*^2^ = 0.32; Std. β = 2.2 × 10^–7^, *R*^2^ = 0.30, respectively) and waist circumference (Std. β = 1.73 × 10^–7^, *R*^2^ = 0.50; Std. β = 0.28, *R*^2^ = 0.48, respectively) (FDR *p* ≤ 0.05). Additional FDR-adjusted significant associations were observed between plasma xylitol (Std. β = 2.20 × 10^–7^, *R*^2^ = 0.26), glutamic acid (Std. β = 0.31, *R*^2^ = 0.30), and trigonelline (Std. β = 0.34, *R*^2^ = 0.28), as well as urinary niacin (Std. β = 2.55 × 10^–7^, *R*^2^ = 0.32), furoylglycine (Std. β = 8.94 × 10^–8^, *R*^2^ = 0.29), and methyluric acid (Std. β = 0.34, *R*^2^ = 0.28), with SCORE (FDR *p* ≤ 0.05). A negative association was observed between plasma cinnamoylglycine with HbA1c (Std. β = −0.27, *R*^2^ = 0.36, FDR *p* ≤ 0.05). No FDR-adjusted significant associations were observed between metabolites with plasma lipids, glucose, or blood pressure.

## Discussion

### FIBs Identified for the Habitual Intake of Individual Fermented Foods

In the current work, we aimed to identify FIBs for fermented foods consumed in the habitual Dutch adult diet, which included coffee, wine, beer, whole-grain bread, white bread, cheese, yoghurt, and cocoa. A total of 12 plasma and 27 urinary metabolites were identified at level 1 or 2 from nontargeted GC–MS and LC–MS analyses, the majority of which corresponded to the intakes of coffee, wine, and beer (no metabolites were identified for cocoa, white bread, whole-grain bread, cheese, and yoghurt intake). These fermented foods were also coincidentally those with the highest intakes (in g/day) in the Dutch adult diet and span a wide range of intakes, which is conducive for the selection of discriminant metabolites. Several of the most promising FIBs identified for these foods were also previously captured by other nontargeted and targeted studies. For instance, plasma/serum quinate and trigonelline, as well as urinary niacin, furoylglycine, catechol, and methyluric and dimethyluric acids, have been previously reported as candidate FIBs of habitual coffee intake.^55−66^ Out of the metabolites identified for wine intake, hydroxy(*iso*)butyric acid has been previously detected in serum after long-term (>4 weeks) wine intake,^67^ tartaric acid in urine following acute wine intake,^68,69^ and urinary 4-hydroxybenzeneacetic acid in urine following both acute and long-term (>4 weeks) wine intake.^67,70−73^ The detection of these previously identified FIBs in our free-living population further supports their status as reliable indicators of the habitual intake of these fermented foods.

Additionally, several metabolites were identified for coffee, wine, and beer intake which have not been previously reported. For instance, we found urinary 3-deoxy-d-*ribo*-hexonic acid γ-lactone to be discriminant for coffee intake. This compound is a degradative product of glucose produced during the Maillard reaction,^74^ which could have formed during coffee brewing. For wine intake, plasma xylitol, plasma/urinary erythritol, and urinary glucuronic acid, citramalate, 2,3-dihydroxybutanoic acid, arabinofuranose, and ethyl α-d-glucopyranoside were identified as potential FIBs. Furthermore, for beer intake, plasma l-cysteine, urinary 2,3-dihydroxypropyl phosphoric acid, 4-hydroxybenzeneacetic acid, d-lactose, 2,3-dihydroxybutanoic acid, and ethyl α-d-glucopyranoside were identified. While not detected previously in biofluids, almost all of these metabolites have been detected or quantified in the associated foods themselves. Erythritol (a natural sugar alcohol) has been previously detected in multiple fermented foods, including wine, beer, sake, coffee, cheese, and soy sauce.^56,75−77^ Interestingly, erythritol can be produced by microorganisms (*e.g.*, *Penicillium* sp. used in the ripening of cheese).^15,77^ Similarly, citramalate (a microbial metabolite that is found to be a byproduct of *Saccharomyces*, *Propionibacterium acnes*, and *Aspergillus niger*) has been detected in red wine.^78,79^ The detection of these metabolites in the plasma and urine metabolomes of free-living individuals consuming these fermented foods indicates that “fermentation-dependent” metabolites could act as powerful complementary FIBs (in addition to other FIBs originating from the raw food substrate) to improve the accuracy of dietary assessment of fermented foods in future studies. Thus, further validation studies are required in order to confirm the robustness and reliability of these newly identified FIBs (e.g., from the “identification cohort”) in a separate population (e.g., “validation cohort”).

One major challenge to the validation of single FIBs relates to their nonspecificity for a particular food. Indeed, the vast majority of metabolites identified for the intake of coffee, wine, and beer as described above have also been detected in biofluids following the consumption of other foods. For instance, plasma/serum quinic acid and urinary furoylglycine have also been identified for habitual cocoa intake,^80,81^ while methyluric and dimethyluric acids, being caffeine metabolites, have naturally also been identified for the intake of caffeinated foods (*i.e.*, cocoa and tea).^81,82^ The phenolic 4-hydroxybenzeneacetic acid corresponding to wine and beer intake has also been detected in urine after acute bread intake^83^ and in serum after long-term coffee intake.^56^ Furthermore, tartaric acid, a fairly specific FIB for wine intake, has also been identified in urine following acute and long-term bread intake^83^ as well as acute beer intake.^84^ The limitations of using these single metabolites as FIBs could be circumvented by developing reliable multimarker panels.^5^ On the other hand, for fermented foods, nonspecific markers shared between different foods could also be useful for indicating common raw materials, fermentation processes (*e.g.*, lactic, acetic, alcoholic, or alkaline fermentation), and/or fermentation with common microorganisms (*e.g.*, lactic acid bacteria, with yeast). This work could be further extended by pathway analyses for the identified compounds, which could aid greatly in understanding their relationships to other metabolites and to human health.

### FIBs Identified for the Intake of Groups of Fermented Foods

In the current work, we also explored using dry matter as a novel method to unify individual fermented foods with similar qualities into fermented food groups. Several groups were generated: total FB (comprising coffee, wine, and beer), total FCG (whole-grain and white bread), and total FD (cheese and yoghurt). By far, the largest number of identified metabolites were discriminant for the intake of total FB; however, the significance of the majority of these metabolites appeared to be largely driven by the individual beverages under this group. A few metabolites (plasma 2-hydroxybutyric acid, *trans*-aconitic acid, l-phenylalanine, and isoleucine; urinary guaiacol, 3-hydroxyhippuric acid, and 3,4-dihydroxyhydrocinnamic acid) appeared to be uniquely discriminant for total FBs. One metabolite was identified for total FCG intake (urinary glyceryl–glycoside ether). This metabolite has not been identified as a FIB previously and needs to be validated in further studies. No metabolites were identified for the intakes of cocoa or total FD, which could be due to the low or inconsistent intake of these foods (in the case of cocoa), or the discriminant metabolites being also of endogenous origin and thus influenced more heavily by human metabolism (in the case of fermented dairy).

While this is the first study to identify FIBs in the context of fermented food groups, this is also an area in need of further development. We formed the fermented food groups based on the groups for which the FFQ was previously validated for.^8,15^ Evidently, there could be other strategies to group fermented foods, which could reveal different sets of FIBs. For instance, fermented foods could be grouped based on a common fermentation process (*e.g.*, lactic fermented foods and yeast-fermented foods), which may further reveal fermentation-dependent FIBs. Unfortunately, we did not have access to information on the fermenting microorganisms in order to group fermented foods according to this strategy. In addition, while we did not consider a total fermented food group in the current study, a total intake group might be worth exploring in future which would be highly relevant to examine the health impacts of a dietary pattern of fermented foods.

### Methodological Considerations for the Identification and Stability of the Biomarkers

Although the primary aim of this study was to identify FIBs for the habitual intake of fermented foods, this work also contributes several methodological insights. First, to comprehensively capture the metabolome for FIB identification, we analyzed two biosamples (plasma and urine) using two analytical platforms (GC–MS and LC–MS). The 24 h urine samples were anticipated to better capture FIBs than plasma collected under fasting conditions, as depending on the speed of metabolism, the metabolite may not be detected even several hours after ingestion in plasma. Indeed, a larger number of urinary metabolites were significant and identified. Still, the number of significant urinary metabolites likely represented a smaller fraction of the total significant (and biologically relevant) metabolites in urine but which are present at relatively low concentrations due to dilution (a necessary step to ensure metabolites are measured within the linear range of the MS instruments). In addition, there could be differences in the metabolism of different metabolites that influence the detection of potential FIBs (*i.e.*, not all metabolites are eliminated in urine). A combination of these factors may explain why only one identified metabolite (erythritol for wine intake) overlapped between plasma and urine.

We also exploited a combination of univariate and multivariate statistical tests to identify the most discriminant FIBs—a strategy that has been explored by an increasing number of groups.^85−87^ While the results of univariate and multivariate analyses are not always congruent, the use of both statistical approaches can generate complementary sets of FIBs. However, the results should be interpreted within the statistical framework from which they have been generated.^88^ Additionally, we ran our analyses in positive mode to make use of the optimized settings by which the widest range of metabolites are expected to ionize and thus be detected. However, we recognize that running the same samples through negative mode (which was not possible due to time and economic constraints) could have been beneficial for expanding the detection of different metabolites and aid in metabolite identification.

Additionally, we investigated the stability of the identified FIBs with increasing time frames between biosample collection and completion of self-reported dietary assessment. This was a necessary analysis since the biosample collection did not occur at the same time as the dietary assessment and could be a source of variability. Importantly, we observed excellent stability (correlation coefficient and significance were maintained) with increasing time from biosample collection to the FFQ completion (within 14 d, 30 d, 180 d, and all) for almost all of the identified FIBs. The driving force for this stability could be the larger numbers of participants in longer time frames (affording more statistical power). Moreover, these results could indicate that the FFQ used to collect information on self-reported fermented food intake in this study is fairly robust and/or that the diets of this population are very stable.

### Associations between Identified FIBs and CMD Risk Parameters

We further examined associations between the identified FIBs with several CMD risk factors as a preliminary analysis to unravel the complex relationships between fermented food consumption and cardiometabolic health. Of the 39 metabolites identified, 7 were positively and 1 was negatively significantly associated with CMD risk factors after adjustment for multiple comparisons. All associations were weak, which may be attributed to the relatively healthy study population that may not have provided the gradient of CMD risk required to observe a large effect size. Thus, these associations need to be confirmed in larger, prospective cohorts or populations with a more distinctive divide between low and high CMD risk. Nonetheless, some of the associations we found have been reported in the literature. For instance, plasma glutamic acid has been positively associated with obesity, particularly with metabolically unhealthy obese phenotypes.^89^ Other associations are more contested. In some studies, the consumption of non-nutritive sweeteners (which includes xylitol) has been shown to increase weight and waist circumference, as well as the incidence of obesity, hypertension, metabolic syndrome, type II diabetes, and cardiovascular events.^90^ However, several recent systematic reviews have also revealed that the use of non-nutritive sweeteners (instead of sugar) reduces energy intake as well as body weight.^91,92^ In all studies, the distinct effects of xylitol (compared to other non-nutritive sweeteners) as well as the underlying mechanisms behind these associations have yet to be verified. Similarly, while we observed significant positive associations between urinary niacin and plasma trigonelline with overall CMD risk (SCORE), this is not in line with the literature for specific CMD risk factors. Niacin is used as pharmacotherapy to prevent cardiovascular disease by lowering cholesterol levels in blood.^93^ In mechanistic studies, trigonelline has been shown to improve insulin sensitivity by interfering with NADPH oxidase gene expression of pathways and mitochondrial electron chain transport.^94^ Given these conflicting findings, further studies in larger, prospective cohorts are needed to clarify these associations and examine whether they play an intermediate role in CMD.

## Supporting Information Available

Classification of fermented foods from NQplus; summary of the significant metabolites for univariate and multivariate statistical analyses; list of suppliers of analytical standards; metabolites prioritized for identification from GC–MS and LC–MS; Spearman’s correlations between identified metabolites and self-reported fermented food intakes (PDF)
Results of multiple linear regression (Table S7) (XLSX)

## Abbreviations

CMD: cardiometabolic disease
FB: fermented beverage
FCGs: fermented cereals and grains
FD: fermented dairy
FDR: false discovery rate
FFQ: food frequency questionnaire
FIB: food intake biomarker
MetS: metabolic syndrome
MSI: Metabolomics Standards Initiative
NDARD: National Dietary Assessment Reference Database
NQplus: Nutritional Questionnaire plus
PCA: principal component analysis
PLS-DA: partial least-square discriminant analysis
QC: quality control
SCORE: Systematic COronary Risk Evaluation
VIP: variable importance in projection
