# Supplementary material for: Identifying Plasma and Urinary Biomarkers of Fermented Food Intake and Their Associations with Cardiometabolic Health in a Dutch Observational Cohort
Source: J Agric Food Chem. 2023 Feb 28;71(10):4426–39. doi: 10.1021/acs.jafc.2c05669 (PMC10021015; doi:10.1021/acs.jafc.2c05669)
Supplement: Supplementary file 1 — jf2c05669_si_001.pdf [file jf2c05669_si_001.pdf]

Supporting Information

Identifying plasma and urinary biomarkers of fermented food intake and their associations with cardiometabolic health in a Dutch observational cohort

Katherine J. Li[1,2], Kathryn J. Burton-Pimentel[2], Elske M. Brouwer-Brolsma[1], Carola Blaser[2], René Badertscher[2], Grégory Pimentel[2], Reto Portmann[2], Edith J.M. Feskens[1], Guy Vergères[2]*

[1] Division of Human Nutrition and Health, Department of Agrotechnology and Food Science, Wageningen University & Research, P.O. Box 17, 6700 AA Wageningen, The Netherlands

[2] Agroscope, Schwarzenburgstrasse 161, CH-3003 Bern, Switzerland

* Corresponding Author

Agroscope

Schwarzenburgstrasse 161, CH-3003 Bern

Tel. +41 (0)58 463 81 54

guy.vergeres@agroscope.admin.ch

Table S1. Classification of fermented foods from the NQplus food frequency questionnaire		
**Food item for classification**	**Fermented food group**	**Fermented food**
Beer	Beverages	Beer
Low-alcohol Beer	Beverages	Beer
Coffee	Beverages	Coffee
Wine, other	Beverages	Wine
Wine, red	Beverages	Wine
Brown bread	Cereals/grains	Bread, brown
Brown bread (slices)	Cereals/grains	Bread, brown
Rye bread	Cereals/grains	Bread, rye
Biscuit (bread), white	Cereals/grains	Bread, white
Bread with raisins	Cereals/grains	Bread, white
White bread	Cereals/grains	Bread, white
White bread (slices)	Cereals/grains	Bread, white
Biscuit (bread), whole wheat	Cereals/grains	Bread, wholegrain
Multiple grain bread	Cereals/grains	Bread, wholegrain
Multiple grain bread (slices)	Cereals/grains	Bread, wholegrain
Whole wheat bread (slices)	Cereals/grains	Bread, wholegrain
Cake / pastry	Cereals/grains	Pastry
Croissants	Cereals/grains	Pastry
Chocolate spread	Cocoa-based products	Chocolate
Milk chocolate	Cocoa-based products	Chocolate
Pure chocolate	Cocoa-based products	Chocolate
White chocolate	Cocoa-based products	Chocolate
Buttermilk	Dairy	Buttermilk
Low-fat cheese ($20^+/30^+$)	Dairy	Cheese
Regular cheese (40^+)	Dairy	Cheese
Regular cheese (48^+)	Dairy	Cheese
Cheese as snack	Dairy	Cheese
Cheese with hot meal	Dairy	Cheese
Fat luxury cheese	Dairy	Cheese
Less-fat luxury cheese	Dairy	Cheese
Unknown cheese	Dairy	Cheese
(Fruit) quark with breakfast	Dairy	Quark
Full (fruit) yogurt	Dairy	Yogurt
Full yogurt	Dairy	Yogurt
Semi-skim (fruit) yogurt	Dairy	Yogurt
Semi-skim yogurt	Dairy	Yogurt
Skim (fruit) yogurt	Dairy	Yogurt
Skim yogurt	Dairy	Yogurt
Unknown yogurt	Dairy	Yogurt

NA, not applicable.

Table S2. Summary of the significant features across univariate and multivariate statistical analyses for selection of the most discriminant compounds[a]												
				Spearman's correlation		Kruskal-Wallis		Random Forest				Number
Platform	Biosample	Fermented foods	Internal identifier	r	FDR-adjuted *p*-value	(FDR-adjusted *p*-value)	PLS-DA (VIP score)	Variable Importance	OBB	Accuracy	*p*-value	of sig. tests
GC-MS	Plasma	Beer	Compound_43	0.238	0.045	NA	2.961	NA	NA	NA	NA	2
GC-MS	Plasma	Beer (dry)	Compound_43	0.238	0.044	NA	2.961	NA	NA	NA	NA	2
GC-MS	Plasma	Coffee	Compound_210	0.427	0.000	0.000	5.786	0.020	0.518	0.491	0.018	4
GC-MS	Plasma	Coffee (dry)	Compound_210	0.427	0.000	0.000	5.786	0.020	0.518	0.491	0.018	4
GC-MS	Plasma	Total FB	Compound_210	0.438	0.000	0.000	5.075	0.024	0.500	0.509	0.009	4
GC-MS	Plasma	Total FB	Compound_2651	0.322	0.003	NA	1.369	NA	NA	NA	NA	2
GC-MS	Plasma	Total FB	Compound_55	NA	NA	NA	2.083	0.003	0.500	0.509	0.009	2
GC-MS	Plasma	Total FB (dry)	Compound_171	0.253	0.031	NA	3.933	NA	NA	NA	NA	2
GC-MS	Plasma	Total FB (dry)	Compound_210	0.259	0.027	0.036	3.366	NA	NA	NA	NA	3
GC-MS	Plasma	Total FB (dry)	Compound_31	0.283	0.008	0.012	3.683	NA	NA	NA	NA	3
GC-MS	Plasma	Total FB (dry)	Compound_43	0.278	0.010	0.036	4.234	NA	NA	NA	NA	3
GC-MS	Plasma	Total FB (dry)	Compound_96	0.352	0.000	0.001	4.303	NA	NA	NA	NA	3
GC-MS	Plasma	White bread (dry)	Compound_48	NA	NA	NA	1.778	0.001	0.589	0.491	0.018	2
GC-MS	Plasma	Wine	Compound_21	0.344	0.000	NA	3.280	NA	NA	NA	NA	2
GC-MS	Plasma	Wine	Compound_96	0.319	0.002	NA	3.883	NA	NA	NA	NA	2
GC-MS	Plasma	Wine (dry)	Compound_21	0.338	0.001	NA	3.072	NA	NA	NA	NA	2
GC-MS	Plasma	Wine (dry)	Compound_96	0.315	0.003	0.018	3.732	NA	NA	NA	NA	3
GC-MS	Urine	Beer	Compound_146	0.232	0.028	0.025	1.842	NA	NA	NA	NA	3
GC-MS	Urine	Beer	Compound_23	NA	NA	0.009	2.117	NA	NA	NA	NA	2
GC-MS	Urine	Beer	Compound_278	0.286	0.003	0.025	2.346	NA	NA	NA	NA	3
GC-MS	Urine	Beer	Compound_4970	0.230	0.028	NA	1.705	NA	NA	NA	NA	2
GC-MS	Urine	Beer	Compound_58	0.215	0.050	0.011	2.046	NA	NA	NA	NA	3
GC-MS	Urine	Beer	Compound_63	NA	NA	0.019	1.707	NA	NA	NA	NA	2
GC-MS	Urine	Beer (dry)	Compound_146	0.232	0.028	0.025	1.842	NA	NA	NA	NA	3
GC-MS	Urine	Beer (dry)	Compound_23	NA	NA	0.009	2.117	NA	NA	NA	NA	2
GC-MS	Urine	Beer (dry)	Compound_278	0.286	0.003	0.025	2.346	NA	NA	NA	NA	3
GC-MS	Urine	Beer (dry)	Compound_4970	0.231	0.028	NA	1.705	NA	NA	NA	NA	2
GC-MS	Urine	Beer (dry)	Compound_58	0.215	0.050	0.011	2.046	NA	NA	NA	NA	3
GC-MS	Urine	Beer (dry)	Compound_63	NA	NA	0.019	1.707	NA	NA	NA	NA	2

Table S2. Summary of the significant features across univariate and multivariate statistical analyses for selection of the most discriminant compounds[a]												
				Spearman's correlation		Kruskal-Wallis		Random Forest				Number
			Internal		FDR-adjuted	(FDR-adjusted	PLS-DA	Variable				of sig.
Platform	Biosample	Fermented foods	identifier	r	*p*-value	*p*-value)	(VIP score)	Importance	OBB	Accuracy	*p*-value	tests
GC-MS	Urine	Coffee	Compound_17	0.334	0.000	0.000	1.922	NA	NA	NA	NA	3
GC-MS	Urine	Coffee	Compound_20	0.457	0.000	0.000	2.432	0.005	0.488	0.537	0.002	4
GC-MS	Urine	Coffee	Compound_23	NA	NA	0.028	1.617	NA	NA	NA	NA	2
GC-MS	Urine	Coffee	Compound_2451	0.401	0.000	0.000	2.171	0.004	0.488	0.537	0.002	4
GC-MS	Urine	Coffee	Compound_247	NA	NA	NA	1.145	0.001	0.488	0.537	0.002	2
GC-MS	Urine	Coffee	Compound_29	0.409	0.000	0.000	2.273	0.005	0.488	0.537	0.002	4
GC-MS	Urine	Coffee	Compound_2992	0.280	0.001	0.002	1.502	0.001	0.488	0.537	0.002	4
GC-MS	Urine	Coffee	Compound_3094	NA	NA	0.000	2.100	0.001	0.488	0.537	0.002	3
GC-MS	Urine	Coffee	Compound_56	0.380	0.000	0.000	2.040	0.004	0.488	0.537	0.002	4
GC-MS	Urine	Coffee (dry)	Compound_17	0.334	0.000	0.000	1.922	NA	NA	NA	NA	3
GC-MS	Urine	Coffee (dry)	Compound_20	0.457	0.000	0.000	2.432	0.005	0.488	0.537	0.002	4
GC-MS	Urine	Coffee (dry)	Compound_23	NA	NA	0.028	1.617	NA	NA	NA	NA	2
GC-MS	Urine	Coffee (dry)	Compound_2451	0.401	0.000	0.000	2.171	0.004	0.488	0.537	0.002	4
GC-MS	Urine	Coffee (dry)	Compound_247	NA	NA	NA	1.145	0.001	0.488	0.537	0.002	2
GC-MS	Urine	Coffee (dry)	Compound_29	0.409	0.000	0.000	2.273	0.005	0.488	0.537	0.002	4
GC-MS	Urine	Coffee (dry)	Compound_2992	0.280	0.001	0.002	1.502	0.001	0.488	0.537	0.002	4
GC-MS	Urine	Coffee (dry)	Compound_3094	NA	NA	0.000	2.100	0.001	0.488	0.537	0.002	3
GC-MS	Urine	Coffee (dry)	Compound_56	0.380	0.000	0.000	2.040	0.004	0.488	0.537	0.002	4
GC-MS	Urine	Total FB	Compound_134	NA	NA	0.011	1.402	NA	NA	NA	NA	2
GC-MS	Urine	Total FB	Compound_146	0.293	0.000	0.004	1.731	NA	NA	NA	NA	3
GC-MS	Urine	Total FB	Compound_148	0.203	0.018	NA	1.492	NA	NA	NA	NA	2
GC-MS	Urine	Total FB	Compound_17	0.329	0.000	0.000	1.947	NA	NA	NA	NA	3
GC-MS	Urine	Total FB	Compound_185	0.224	0.007	NA	1.321	NA	NA	NA	NA	2
GC-MS	Urine	Total FB	Compound_20	0.427	0.000	0.000	2.299	NA	NA	NA	NA	3
GC-MS	Urine	Total FB	Compound_23	NA	NA	0.000	2.118	NA	NA	NA	NA	2
GC-MS	Urine	Total FB	Compound_245	0.203	0.018	NA	1.438	NA	NA	NA	NA	2
GC-MS	Urine	Total FB	Compound_2451	0.370	0.000	0.000	2.021	NA	NA	NA	NA	3
GC-MS	Urine	Total FB	Compound_278	0.250	0.002	0.014	2.009	NA	NA	NA	NA	3
GC-MS	Urine	Total FB	Compound_29	0.347	0.000	0.000	2.062	NA	NA	NA	NA	3

Table S2. Summary of the significant features across univariate and multivariate statistical analyses for selection of the most discriminant compounds[a]												
				Spearman's correlation		Kruskal-Wallis		Random Forest				Number
Platform	Biosample	Fermented foods	Internal identifier	r	FDR-adjuted *p*-value	(FDR-adjusted *p*-value)	PLS-DA (VIP score)	Variable Importance	OBB	Accuracy	*p*-value	of sig. tests
GC-MS	Urine	Total FB	Compound_2992	0.269	0.001	0.016	1.312	NA	NA	NA	NA	3
GC-MS	Urine	Total FB	Compound_3094	NA	NA	0.000	1.119	NA	NA	NA	NA	2
GC-MS	Urine	Total FB	Compound_4970	0.247	0.002	NA	1.580	NA	NA	NA	NA	2
GC-MS	Urine	Total FB	Compound_56	0.369	0.000	0.000	2.201	NA	NA	NA	NA	3
GC-MS	Urine	Total FB	Compound_58	0.259	0.001	0.000	1.587	NA	NA	NA	NA	3
GC-MS	Urine	Total FB	Compound_63	0.263	0.001	0.002	1.389	NA	NA	NA	NA	3
GC-MS	Urine	Total FB (dry)	Compound_134	NA	NA	0.016	1.627	0.001	0.401	0.593	0.000	3
GC-MS	Urine	Total FB (dry)	Compound_146	0.378	0.000	0.000	1.958	0.002	0.401	0.593	0.000	4
GC-MS	Urine	Total FB (dry)	Compound_156	0.281	0.001	0.016	1.629	NA	NA	NA	NA	3
GC-MS	Urine	Total FB (dry)	Compound_20	0.259	0.002	NA	1.586	0.001	0.401	0.593	0.000	3
GC-MS	Urine	Total FB (dry)	Compound_23	NA	NA	0.000	2.349	0.006	0.401	0.593	0.000	3
GC-MS	Urine	Total FB (dry)	Compound_278	0.417	0.000	0.000	2.802	0.021	0.401	0.593	0.000	4
GC-MS	Urine	Total FB (dry)	Compound_3874	0.268	0.001	0.004	1.850	0.001	0.401	0.593	0.000	4
GC-MS	Urine	Total FB (dry)	Compound_4970	0.260	0.002	NA	1.695	0.000	0.401	0.593	0.000	3
GC-MS	Urine	Total FB (dry)	Compound_52	NA	NA	0.016	1.665	0.001	0.401	0.593	0.000	3
GC-MS	Urine	Total FB (dry)	Compound_56	0.205	0.019	NA	1.464	NA	NA	NA	NA	2
GC-MS	Urine	Total FB (dry)	Compound_58	0.230	0.007	NA	1.184	NA	NA	NA	NA	2
GC-MS	Urine	Total FB (dry)	Compound_63	0.308	0.000	0.001	2.232	0.001	0.401	0.593	0.000	4
GC-MS	Urine	Total FB (dry)	Compound_96	NA	NA	NA	1.508	0.001	0.401	0.593	0.000	2
GC-MS	Urine	Total FCG	Compound_19	0.383	0.000	0.000	NA	NA	NA	NA	NA	2
GC-MS	Urine	Total FCG (dry)	Compound_19	0.385	0.000	0.000	NA	NA	NA	NA	NA	2
GC-MS	Urine	Wine	Compound_146	0.283	0.001	0.002	2.033	NA	NA	NA	NA	3
GC-MS	Urine	Wine	Compound_156	0.405	0.000	0.000	2.498	NA	NA	NA	NA	3
GC-MS	Urine	Wine	Compound_164	NA	NA	0.044	1.438	NA	NA	NA	NA	2
GC-MS	Urine	Wine	Compound_198	0.235	0.015	NA	1.869	NA	NA	NA	NA	2
GC-MS	Urine	Wine	Compound_278	0.224	0.017	0.040	1.614	NA	NA	NA	NA	3
GC-MS	Urine	Wine	Compound_3874	0.417	0.000	0.000	3.053	NA	NA	NA	NA	3
GC-MS	Urine	Wine	Compound_96	0.338	0.000	0.000	2.665	NA	NA	NA	NA	3
GC-MS	Urine	Wine (dry)	Compound_102	NA	NA	NA	1.639	0.001	0.420	0.463	0.032	2

Table S2. Summary of the significant features across univariate and multivariate statistical analyses for selection of the most discriminant compounds[a]												
				Spearman's correlation		Kruskal-Wallis		Random Forest				Number
Platform	Biosample	Fermented foods	Internal identifier	r	FDR-adjuted *p*-value	(FDR-adjusted *p*-value)	PLS-DA (VIP score)	Variable Importance	OBB	Accuracy	*p*-value	of sig. tests
GC-MS	Urine	Wine (dry)	Compound_146	0.280	0.001	0.002	2.069	0.003	0.420	0.463	0.032	4
GC-MS	Urine	Wine (dry)	Compound_156	0.406	0.000	0.000	2.534	0.006	0.420	0.463	0.032	4
GC-MS	Urine	Wine (dry)	Compound_164	NA	NA	0.036	1.494	0.002	0.420	0.463	0.032	3
GC-MS	Urine	Wine (dry)	Compound_198	0.234	0.016	NA	1.905	NA	NA	NA	NA	2
GC-MS	Urine	Wine (dry)	Compound_278	0.226	0.017	0.036	1.622	0.001	0.420	0.463	0.032	4
GC-MS	Urine	Wine (dry)	Compound_3874	0.415	0.000	0.000	3.066	0.005	0.420	0.463	0.032	4
GC-MS	Urine	Wine (dry)	Compound_96	0.332	0.000	0.000	2.680	NA	NA	NA	NA	3
LC-MS	Plasma	Coffee	Compound_3276	NA	NA	0.034	1.962	0.005	0.532	0.627	0.000	3
LC-MS	Plasma	Coffee	Compound_3903	NA	NA	NA	1.147	0.001	0.532	0.627	0.000	2
LC-MS	Plasma	Coffee (dry)	Compound_3276	NA	NA	0.034	1.962	0.005	0.532	0.627	0.000	3
LC-MS	Plasma	Coffee (dry)	Compound_3903	NA	NA	NA	1.147	0.001	0.532	0.627	0.000	2
LC-MS	Plasma	Wine	Compound_7825	0.597	0.000	0.018	NA	NA	NA	NA	NA	2
LC-MS	Plasma	Wine (dry)	Compound_7825	0.599	0.000	0.018	NA	NA	NA	NA	NA	2
LC-MS	Urine	Coffee	Compound_3885	0.477	0.000	0.000	3.101	0.000	0.400	0.463	0.032	4
LC-MS	Urine	Coffee	Compound_4443	0.496	0.000	0.000	3.082	0.000	0.400	0.463	0.032	4
LC-MS	Urine	Coffee (dry)	Compound_3885	0.477	0.000	0.000	3.101	0.000	0.400	0.463	0.032	4
LC-MS	Urine	Coffee (dry)	Compound_4443	0.496	0.000	0.000	3.082	0.000	0.400	0.463	0.032	4

FB, fermented beverages; FCG, fermented cereals and grains; GC-MS, gas chromatography mass spectrometry; LC-MS, liquid chromatography mass spectrometry; NA, not applicable.

[a] Only features that were significant across at least two of the four statistical tests (and thus prioritized for identification) are included in this table. Feature information (e.g., m/z, retention time) is provided in Tables S4 and S5.

**Table S3. List of suppliers of analytical standards**	
**Compound**	**Supplier**
Erythritol	MSML kit (IROA Technologies, LLC, Bolton, MA; Gainesville, FL)
2-Hydroxybutyric acid	MSML kit (IROA Technologies, LLC, Bolton, MA; Gainesville, FL)
L-Cysteine	MSML kit (IROA Technologies, LLC, Bolton, MA; Gainesville, FL)
Dodecanoic acid	Merck, Darmstadt, Germany
Xylitol	MSML kit (IROA Technologies, LLC, Bolton, MA; Gainesville, FL)
trans-Aconitic acid	MSML kit (IROA Technologies, LLC, Bolton, MA; Gainesville, FL)
D-Quinate	MSML kit (IROA Technologies, LLC, Bolton, MA; Gainesville, FL)
L-Phenylalanine	MSML kit (IROA Technologies, LLC, Bolton, MA; Gainesville, FL)
L-Isoleucine	Merck, Darmstadt, Germany
D-Psicose	MSML kit (IROA Technologies, LLC, Bolton, MA; Gainesville, FL)
Glycine	Merck, Darmstadt, Germany
D-Gluconate	MSML kit (IROA Technologies, LLC, Bolton, MA; Gainesville, FL)
Guaiacol	Sigma-Aldrich, Switzerland
D-Lactose	Merck, Darmstadt, Germany
Niacin	MSML kit (IROA Technologies, LLC, Bolton, MA; Gainesville, FL)
Catechol	Sigma-Aldrich, Switzerland
Citramalate	MSML kit (IROA Technologies, LLC, Bolton, MA; Gainesville, FL)
Tartaric acid	MSML kit (IROA Technologies, LLC, Bolton, MA; Gainesville, FL)
L-Glutamic acid	MSML kit (IROA Technologies, LLC, Bolton, MA; Gainesville, FL)
Trigonelline	MSML kit (IROA Technologies, LLC, Bolton, MA; Gainesville, FL)
(S)-3-Hydroxyisobutyric acid	Sigma-Aldrich, Switzerland, ≥96.0% (sodium salt)
1-Methyluric acid	Toronto Research Chemicals, Toronto, Canada
1,3-Dimethyluric acid	Toronto Research Chemicals, Toronto, Canada
Cinnamoylglycine	Combi-Blocks, Inc., San Diego, FL

Table S4. Features prioritized for identification from GC-MS (37 plasma, 75 urine)										
**Internal identifier**	**Identification**	**Fermented food(s)**	**RT (min)**	**Quantifier Ion**	**Qualifier Ion**	**Ratio (Quant/Qual)**	**RSD QC**	**RI sample**	**RI reference**	**ID level**
**Plasma[a]**										
Compound_21	Erythritol (4TMS)	Wine, Wine (dry)	25.01	217	205	65	36.6	1491	1494	1
Compound_31	2-Hydroxybutyric acid (2TMS)	Total FB (dry)	15.72	131	233	7	41.5	1116	1119	1
Compound_43	L-Cysteine (3TMS)	Beer, Beer (dry), Total FB (dry)	25.96	220	218	90	60.6	1548	1548	1
Compound_48	Dodecanoic acid (1TMS)	White bread (dry)	27.48	257	117	75	32.3	1647	1649	1
Compound_96	Xylitol (5TMS)	Total FB (dry), Wine, Wine (dry)	28.2	217	307	35	41.4	1698	1690	1
Compound_171	trans-Aconitic acid (3TMS)	Total FB (dry)	28.62	375	285	62	46.8	1731	1733	1
Compound_210	Quinate (5TMS)	Coffee, Coffee (dry), Total FB, Total FB (dry)	29.91	345	255	45	43.1	1839	1843	1
Compound_2651	L-Phenylalanine (1TMS)	Total FB	26.03	120	146	50	61.4	1552	1550	1
Compound_55	Isoleucine (2TMS)	Total FB	20.5	158	218	22	44.6	1285	1285	1
Compound_73	Unknown	White bread (dry)	35.74	411	290	350	38.6	2444	n.d.	4
Compound_94	Unknown	Beer, Beer (dry), Total FB (dry)	35.96	364	290	100	64.4	2467	n.d.	4
Compound_110	Unknown	Total FB (dry)	31.57	174	n.d.	n.d.	62.9	1992	n.d.	4
Compound_134	Unknown	White bread (dry)	38.32	290	541	15	79.1	2689	n.d.	4
Compound_165	Unknown	Beer, Beer (dry), Total FB (dry)	32.31	383	398	65	57.2	2069	n.d.	4
Compound_173	Unknown	Total FB (dry), Wine, Wine (dry)	30.68	204	n.d.	n.d.	34.2	1907	n.d.	4
Compound_276	Unknown	White bread (dry)	26.5	172	146	240	52.5	1581	n.d.	4
Compound_328	Unknown	White bread (dry)	33.14	345	214	50	65.2	2157	n.d.	4
Compound_611	Unknown	Coffee, Coffee (dry)	26.94	208	192	70	52.0	1609	n.d.	4
Compound_702	Unknown	White bread (dry)	25.13	337	319	50	33.1	1497	n.d.	4
Compound_747	Unknown	Coffee, Coffee (dry)	24.06	302	317	75	38.5	1441	n.d.	4
Compound_823	Unknown	Total FB	27.53	268	181	33	62.6	1651	n.d.	4
Compound_929	Unknown	Coffee, Coffee (dry)	35	221	n.d.	n.d.	70.4	2364	n.d.	4

Table S4. Features prioritized for identification from GC-MS (37 plasma, 75 urine)										
**Internal identifier**	**Identification**	**Fermented food(s)**	**RT (min)**	**Quantifier Ion**	**Qualifier Ion**	**Ratio (Quant/Qual)**	**RSD QC**	**RI sample**	**RI reference**	**ID level**
Compound_1088	Unknown	White bread (dry)	26.87	166	255	220	64.1	1604	n.d.	4
Compound_2349	Unknown	Cocoa, Cocoa (dry)	37.82	512	290	87	49.1	2645	n.d.	4
Compound_104	Unknown	Total FB	35.22	103	232	99	43.1	2390	n.d.	4
Compound_147	Unknown	Total FB (dry)	31.29	266	237	49	36.6	1967	n.d.	4
Compound_166	Unknown	Total FB	29.92	297	140	68	50.2	1840	n.d.	4
Compound_876	Unknown	Wine (dry)	47.27	353	443	28	25.2	3168	n.d.	4
Compound_2273	Unknown	Wine, Wine (dry)	47.27	353	443	28	25.2	3168	n.d.	4
**Urine**[b]										
Compound_17	Catechol (2TMS)	Coffee, Coffee (dry), Total FB	21.22	254	239	45	23.9	1314	1314	1
Compound_20	Niacin (1TMS) (Nicotinate/Vitamin B3)	Coffee, Coffee (dry), Total FB, Total FB (dry)	20.78	180	136	50	54.4	1297	1293	1
Compound_23	D-Psicose (5TMS 1MEOXa)	Beer, Beer (dry), Coffee, Coffee (dry), Total FB, Total FB (dry)	29.81	307	217	106	24.0	1834	1837	1
Compound_52	Glycine (3TMS)	Total FB (dry)	20.89	174	248	20	24.2	1300	1300	1
Compound_96	Erythritol (4TMS)	Total FB (dry), Wine, Wine (dry)	24.97	217	205	60	21.1	1491	1494	1
Compound_156	Tartaric acid (4TMS)	Total FB (dry), Wine, Wine (dry)	27.03	292	219	45	21.6	1619	1621	1
Compound_164	D-Gluconate (6TMS)	Wine, Wine (dry)	31.42	333	292	90	67.8	1982	1986	1
Compound_185	Guaiacol (1TMS)	Total FB	18.79	166	181	32	26.6	1226	1224	1
Compound_198	Citramalate (3TMS)	Wine, Wine (dry)	24.44	247	259	20	23.4	1464	1464	1
Compound_4970	D-Lactose (8TMS 1MEOXb)	Beer, Beer (dry), Total FB, Total FB (dry)	38.18	361	204	168	24.8	2682	2691	1
Compound_19	Glyceryl-glycoside TMS ether	Total FCG, Total FCG (dry)	34.18	337	204	555	22.2	n.d.	n.d.	2
Compound_29	Furoylglycine (1TMS)	Coffee, Coffee (dry), Total FB	27.61	95	169	40	36.1	n.d.	n.d.	2

Table S4. Features prioritized for identification from GC-MS (37 plasma, 75 urine)										
**Internal identifier**	**Identification**	**Fermented food(s)**	**RT (min)**	**Quantifier Ion**	**Qualifier Ion**	**Ratio (Quant/Qual)**	**RSD QC**	**RI sample**	**RI reference**	**ID level**
Compound_56	3-deoxy-2,5,6-tris-O-(TMS)-D-ribo-hexonic acid gamma-lactone	Coffee, Coffee (dry), Total FB, Total FB (dry)	29.02	246	129	150	31.2	1767	n.d.	2
Compound_63	2,3-Dihydroxybutanoic acid (3TMS)	Beer, Beer (dry), Total FB, Total FB (dry)	21.87	292	220	40	21.3	n.d.	n.d.	2
Compound_58	Phosphoric acid, bis(TMS) 2,3-bis[(trimethylsilyl)oxy]propyl ester (2,3-Dihydroxypropyl phosphoric acid (4TMS))	Beer, Beer (dry), Total FB, Total FB (dry)	28.70	357	445	21	22.2	1742	n.d.	2
Compound_102	Glucuronic acid (5TMS 1MEOX)	Wine (dry)	30.74	333	160	43	22.1	1917	n.d.	2
Compound_134	m-Cresol (1TMS)	Total FB, Total FB (dry)	16.67	165	180	43	30.9	1151	n.d.	2
Compound_146	4-Hydroxybenzeneacetic acid (2TMS)	Beer, Beer (dry), Total FB, Total FB (dry), Wine, Wine (dry)	27.27	252	296	90	25.8	1636	n.d.	2
Compound_148	3-Hydroxyhippuric acid, O,O'-bis-TMS	Total FB	32.97	294	193	63	28.3	2143	n.d.	2
Compound_245	3,4-Dihydroxyhydrocinnamic acid (3TMS)	Total FB	30.94	179	267	70	31.6	1936	n.d.	2
Compound_247	D-Fucitol (5TMS)	Coffee, Coffee (dry)	28.80	117	205	67	34.5	1750	n.d.	2
Compound_278	Ethyl α-D-glucopyranoside (4TMS)	Beer, Beer (dry), Total FB, Total FB (dry), Wine, Wine (dry)	30.17	204	217	22	23.8	n.d.	n.d.	2
Compound_3094	2-Keto-l-gluconic acid (5TMS)	Coffee, Coffee (dry), Total FB	28.87	292	333	23	30.1	n.d.	n.d.	2
Compound_3874	Arabinofuranose (4TMS)	Total FB (dry), Wine, Wine (dry)	26.34	217	218	20	26.1	n.d.	n.d.	2
Compound_158	Sugar alcohol	Total FB (dry), Wine, Wine (dry)	27.99	217	205	35	26.1	n.d.	n.d.	3
Compound_2504	Monosaccharide	Coffee, Coffee (dry), Total FB, Total FB (dry)	30.64	258	332	92	25.2	1908	n.d.	3
Compound_2911	Monosaccharide	Total FB (dry), Wine (dry)	27.94	217	307	95	26.8	1683	n.d.	3

Table S4. Features prioritized for identification from GC-MS (37 plasma, 75 urine)										
**Internal identifier**	**Identification**	**Fermented food(s)**	**RT (min)**	**Quantifier Ion**	**Qualifier Ion**	**Ratio (Quant/Qual)**	**RSD QC**	**RI sample**	**RI reference**	**ID level**
Compound_4596	Disaccharide	Coffee, Coffee (dry), Total FB	38.09	319	205	42	27.5	2675	n.d.	3
Compound_14	Unknown (possibly quininic acid)	Coffee, Coffee (dry), Total FB, Total FB (dry)	29.88	345	255	35	15.1	n.d.	n.d.	4
Compound_84	Unknown	Coffee, Coffee (dry), Total FB	31.46	159	217	307	30.2	n.d.	n.d.	4
Compound_111	Unknown	Coffee, Coffee (dry), Total FB, Total FB (dry)	29.83	267	280	31	20.6	1835	n.d.	4
Compound_140	Unknown	Coffee, Coffee (dry), Total FB, Total FB (dry)	30.49	298	226	5	22.2	n.d.	n.d.	4
Compound_2423	Unknown	Total FB (dry), Wine, Wine (dry)	31.20	217	189	9	28.2	1962	n.d.	4
Compound_2451	Unknown	Coffee, Coffee (dry), Total FB	28.19	210	136	61	25.5	1701	n.d.	4
Compound_2494	Unknown	Wine, Wine (dry)	34.88	223	369	38	37.5	2357	n.d.	4
Compound_2606	Unknown	Coffee, Coffee (dry), Total FB	29.84	245	335	11	57.5	1837	n.d.	4
Compound_2621	Unknown	Total FB	31.05	373	358	84	45.9	1947	n.d.	4
Compound_2632	Unknown	Coffee, Coffee (dry), Total FB, Total FB (dry)	28.93	292	293	28	31.2	1760	n.d.	4
Compound_2659	Unknown	Total FB (dry)	29.60	217	305	15	30.5	1815	n.d.	4
Compound_2727	Unknown	Beer, Beer (dry), Total FB, Total FB (dry)	30.94	333	292	23	25.2	1937	n.d.	4
Compound_2746	Unknown	Coffee, Coffee (dry), Total FB	27.43	245	231	32	37.4	n.d.	n.d.	4
Compound_2768	Unknown	Beer, Beer (dry), Coffee, Coffee (dry), Total FB, Total FB (dry)	39.19	375	333	10	25.6	n.d.	n.d.	4

Table S4. Features prioritized for identification from GC-MS (37 plasma, 75 urine)										
**Internal identifier**	**Identification**	**Fermented food(s)**	**RT (min)**	**Quantifier Ion**	**Qualifier Ion**	**Ratio (Quant/Qual)**	**RSD QC**	**RI sample**	**RI reference**	**ID level**
Compound_2904	Unknown	Coffee, Coffee (dry), Total FB	29.79	245	246	22	51.5	1832	n.d.	4
Compound_2992	Unknown	Coffee, Coffee (dry), Total FB	29.73	245	335	13	65.4	1826	n.d.	4
Compound_3015	Unknown	Beer, Beer (dry), Total FB, Total FB (dry), Wine, Wine (dry)	29.37	231	243	20	25.3	n.d.	n.d.	4
Compound_3083	Unknown	Wine (dry)	25.51	217	307	70	27.5	n.d.	n.d.	4
Compound_3102	Unknown	Wine, Wine (dry)	35.21	217	103	6.5	25.9	2392	n.d.	4
Compound_3406	Unknown	Wine	n.d.	n.d.	n.d.	n.d.	n.d.	n.d.	n.d.	4
Compound_3418	Unknown	Wine, Wine (dry)	36.02	388	270	70	47.3	2479	n.d.	4
Compound_3447	Unknown	Wine, Wine (dry)	27.06	288	n.d.	n.d.	22.0	1621	n.d.	4
Compound_3463	Unknown	Coffee, Coffee (dry), Total FB, Total FB (dry)	25.39	143	233	65	36.8	1515	n.d.	4
Compound_3497	Unknown	Wine (dry)	26.96	334	232	73	22.7	1614	n.d.	4
Compound_3503	Unknown	Coffee, Coffee (dry)	30.07	345	346	32	13.1	1857	n.d.	4
Compound_3556	Unknown	Total FB, Total FB (dry)	30.12	297	399	4.3	13.5	1861	n.d.	4
Compound_3583	Unknown	Coffee, Coffee (dry), Total FB	35.15	272	375	145	30.5	n.d.	n.d.	4
Compound_3712	Unknown (possibly quininic acid)	Coffee, Coffee (dry), Total FB, Total FB (dry)	30.84	345	255	27	23.0	n.d.	n.d.	4
Compound_3729	Unknown	Total FB (dry)	35.25	375	180	76	23.1	2398	n.d.	4
Compound_3860	Unknown	Wine, Wine (dry)	26.33	84	58	85	48.0	1572	n.d.	4
Compound_3887	Unknown	Coffee, Coffee (dry), Total FB	39.35	375	361	96	22.4	2774	n.d.	4
Compound_3942	Unknown	Coffee, Coffee (dry), Total FB	28.14	268	240	6	22.0	1697	n.d.	4

Table S4. Features prioritized for identification from GC-MS (37 plasma, 75 urine)										
**Internal identifier**	**Identification**	**Fermented food(s)**	**RT (min)**	**Quantifier Ion**	**Qualifier Ion**	**Ratio (Quant/Qual)**	**RSD QC**	**RI sample**	**RI reference**	**ID level**
Compound_3947	Unknown	Coffee, Coffee (dry), Total FB, Total FB (dry)	35.33	375	376	32	25.1	2406	n.d.	4
Compound_3973	Unknown	Coffee, Coffee (dry), Total FB, Total FB (dry)	37.82	522	507	30	48.2	n.d.	n.d.	4
Compound_4022	Unknown	Coffee, Coffee (dry), Total FB, Total FB (dry)	35.68	254	375	240	25.7	n.d.	n.d.	4
Compound_4186	Unknown	Coffee, Coffee (dry)	28.32	294	272	38	27.3	1711	n.d.	4
Compound_4289	Unknown	Total FB (dry)	34.63	186	346	26	29.3	n.d.	n.d.	4
Compound_4400	Unknown	Coffee, Coffee (dry), Total FB, Total FB (dry), Wine, Wine (dry)	30.99	389	491	15	33.0	n.d.	n.d.	4
Compound_4424	Unknown	Coffee, Coffee (dry), Total FB	31.61	429	430	40	60.3	n.d.	n.d.	4
Compound_4554	Unknown	Coffee, Coffee (dry), Total FB, Total FB (dry), Wine, Wine (dry)	39.24	156	431	18	33.1	n.d.	n.d.	4
Compound_4590	Unknown	Total FB (dry)	21.91	245	246	20	26.6	n.d.	n.d.	4
Compound_4683	Unknown	Beer, Beer (dry), Coffee, Coffee (dry), Total FB, Total FB (dry)	36.77	375	257	21	27.9	n.d.	n.d.	4
Compound_4960	Unknown	Wine, Wine (dry)	23.74	239	284	82	27.7	1426	n.d.	4

FB; total fermented beverages; FCG, fermented cereals/grains; FD, fermented dairy; ID, identification; MEOX, methoxyoxamine; n.d., not determined; QC, quality control; RI, retention index; RSD; relative standard deviation; RT, retention time; TMS, trimethylsilyl.

[a]After identification, 8 plasma features were removed due to high levels in blanks or the compounds were found to be originating from the GC column: Compound_0289 (3,5-Diacetyl-4-methyl-1-phenyl-1,4-dihydropyridine), Compound_1281 (Dodecamethylcyclohexasiloxane), Compound_1321 (Decamethylcyclopentasiloxane), Compound_0273 (Alkane), Compound_0083 (Unknown), Compound_2217 (Unknown), Compound_224 (added decane), and Compound_859 (added sucrose).

[b]After identification, 4 urinary features were removed due to high levels in blanks or the compounds were found to be originating from the GC column: Compound_0123 (Dodecamethylpentasiloxane), Compound_0082 (similar to Lumichrome (2MEOX)), Compound_199 (dodecane, 4,6-dimethyl-), and Compound_2723 (unknown).

Table S5. Features prioritized for identification from LC-MS positive mode (13 plasma, 89 urine)							
**Internal identifier**	**Identification**	**Food**	**RT (min)**	**m/z**	**Adducts**	**Measured neutral mass (Da)**	**ID level**
**Plasma**							
Compound_3276	Trigonelline	Coffee, Coffee (dry)	1.04	138.0540	M+H	-	1[a]
Compound_3903	Glutamic acid	Coffee, Coffee (dry)	0.95	148.0602	M+H	-	1[a]
Compound_7825	Hydroxy(*iso*)butyric acid	Wine, Wine (dry)	1.23	-	M+ACN+Na, 2M+Na, M+ACN+H	104.0483	2[a]
Compound_275	Unknown	Coffee, Coffee (dry)	1.23	-	-	86.0191	4
Compound_4335	Unknown	Coffee, Coffee (dry)	2.26	156.1380	-	-	4
Compound_5688	Unknown	Coffee, Coffee (dry)	3.81	-	-	92.0623	4
Compound_5857	Unknown	Coffee, Coffee (dry)	4.80	188.1279	-	-	4
Compound_6870	Unknown	Coffee, Coffee (dry), Total FB, Total FB (dry)	5.61	211.1433	-	-	4
Compound_8569	Unknown	Total FB (dry)	4.63	246.1694	-	-	4
Compound_11880	Unknown	Coffee, Coffee (dry)	8.55		-	316.2030	4
Compound_12051	Unknown	Coffee, Coffee (dry), Total FB	7.19		-	320.1981	4
Compound_12800	Unknown	Coffee, Coffee (dry)	12.51	339.2495	-	-	4
Compound_17536	Unknown	Coffee, Coffee (dry), Total FB	7.19		-	496.2301	4
**Urine**							
Compound_3885	Methyluric acid	Coffee, Coffee (dry)	3.08	-	M+H, M+Na, 2M+H, 2M+Na, M+ACN+Na	182.0437	1[b]
Compound_4443	Dimethyluric acid	Coffee, Coffee (dry)	3.58	-	M+H, 2M+H	196.0591	1[b]
Compound_4819	Cinnamoylglycine	Wine, Wine (dry)	6.74	-	-	205.0734	1[b]
Compound_363	Substituted oxazole	Total FB	1.47	108.0806	M+H-H2O	-	3
Compound_2564	Substituted oxazole	Coffee, Coffee (dry), Total FB	4.1	150.1273	-	-	3
Compound_4970	Substituted oxazole	Coffee, Coffee (dry), Total FB	2.12	209.1641	M+ACN+H	-	3
Compound_9123	Phenylhydrazone derivative	Wine, Wine (dry)	2.85	-	M+H-H2O, M+Na	266.0994	3
Compound_12011	O-glucuronide	Wine, Wine (dry)	2.8	-	M+H-H2O, M+H	356.0945	3
Compound_14478	Phenolic, possibly glucuronidated metabolite of dihydroxyphenylvalerolactone	Wine, Wine (dry)	6.84	385.1130	-	-	3
Compound_16982	Xanthone	Wine, Wine (dry)	2.57	440.1209	-	-	3

Table S5. Features prioritized for identification from LC-MS positive mode (13 plasma, 89 urine)							
**Internal identifier**	**Identification**	**Food**	**RT (min)**	**m/z**	**Adducts**	**Measured neutral mass (Da)**	**ID level**
Compound_719	Unknown	Total FB	1.58	112.0392	-	-	4
Compound_1837	Unknown	Wine, Wine (dry)	6.75	131.049	-	-	4
Compound_3117	Unknown	Coffee, Coffee (dry), Total FB	3.48	-	-	122.0185	4
Compound_3488	Unknown	Coffee, Coffee (dry)	3.65	174.0908	-	-	4
Compound_5054	Unknown	Coffee, Coffee (dry), Total FB	5.64	211.1434	-	-	4
Compound_5924	Unknown	Total FB (dry)	2.07	-	-	208.094	4
Compound_6159	Unknown	Wine, Wine (dry)	2.12	-	-	253.079	4
Compound_6494	Unknown	Coffee, Coffee (dry), Total FB	3.49	-	-	242.0466	4
Compound_6664	Unknown	Beer, Beer (dry), Total FB (dry)	2.99	-	-	245.0716	4
Compound_7380	Unknown	Wine, Wine (dry)	1.87	259.128	-	-	4
Compound_7794	Unknown	Wine, Wine (dry)	3.11	266.0686	-	-	4
Compound_7802	Unknown	Wine, Wine (dry)	2.87	266.1048	-	-	4
Compound_7959	Unknown	Wine, Wine (dry)	1.73	269.1327	-	-	4
Compound_7960	Unknown	Total FB (dry)	2.08	269.133	-	-	4
Compound_8207	Unknown	Total FB (dry), Wine, Wine (dry)	4.28	273.1435	-	-	4
Compound_8260	Unknown	Wine, Wine (dry)	3.86	274.1178	-	-	4
Compound_8334	Unknown	Wine, Wine (dry)	4.57	275.9918	-	-	4
Compound_8403	Unknown	Wine, Wine (dry)	2.54	277.0097	-	-	4
Compound_8761	Unknown	Wine, Wine (dry)	2.07	283.1132	-	-	4
Compound_9057	Unknown	Total FB	2.95	-	-	287.0633	4
Compound_9076	Unknown	Wine, Wine (dry)	4.4	288.1796	-	-	4
Compound_9190	Unknown	Wine, Wine (dry)	4.09	-	-	272.1258	4
Compound_9333	Unknown	Wine, Wine (dry)	6.21	293.0317	-	-	4
Compound_9361	Unknown	Wine, Wine (dry)	4.09	293.1659	-	-	4
Compound_9769	Unknown	Coffee, Coffee (dry)	3.01	300.0751	-	-	4
Compound_9833	Unknown	Coffee, Coffee (dry), Total FB	3.42	301.1021	-	-	4
Compound_10030	Unknown	Total FB (dry), Wine, Wine (dry)	2.83	304.1746	-	-	4
Compound_10055	Unknown	Wine (dry)	6.75	-	-	303.99	4
Compound_10148	Unknown	Wine, Wine (dry)	3.22	306.1533	-	-	4

Table S5. Features prioritized for identification from LC-MS positive mode (13 plasma, 89 urine)							
**Internal identifier**	**Identification**	**Food**	**RT (min)**	**m/z**	**Adducts**	**Measured neutral mass (Da)**	**ID level**
Compound_10198	Unknown	Total FB (dry)	2.08	307.0779	-	-	4
Compound_10249	Unknown	Total FB (dry)	2.07	308.0183	-	-	4
Compound_10310	Unknown	Wine, Wine (dry)	4	309.0952	-	-	4
Compound_10609	Unknown	Wine, Wine (dry)	4.92	-	-	331.0717	4
Compound_10822	Unknown	Total FD	5.53	317.2423	-	-	4
Compound_11010	Unknown	Total FB (dry)	2.07	320.9971	-	-	4
Compound_11641	Unknown	Total FB (dry), Wine, Wine (dry)	3.81	332.1332	-	-	4
Compound_11724	Unknown	Wine, Wine (dry)	3.72	-	-	351.0369	4
Compound_11886	Unknown	Total FB (dry), Wine, Wine (dry)	2.08	337.0315	-	-	4
Compound_11901	Unknown	Wine, Wine (dry)	5.83	337.1298	-	-	4
Compound_12013	Unknown	Wine, Wine (dry)	1.98	-	-	356.0948	4
Compound_12134	Unknown	Total FB	4.1	-	-	323.0458	4
Compound_12196	Unknown	Beer, Beer (dry)	1.87	-	-	324.1196	4
Compound_12309	Unknown	Total FB	6.42	-	-	326.0993	4
Compound_12414	Unknown	Total FB (dry), Wine, Wine (dry)	3.81	346.1121	-	-	4
Compound_12553	Unknown	Wine (dry)	3.72	349.0702	-	-	4
Compound_12669	Unknown	Total FB (dry)	3.79	351.0676	-	-	4
Compound_12954	Unknown	Wine, Wine (dry)	3.83	356.1529	-	-	4
Compound_13124	Unknown	Coffee, Coffee (dry), Total FB	2.97	-	-	358.0751	4
Compound_13285	Unknown	Wine, Wine (dry)	4.71	362.065	-	-	4
Compound_13287	Unknown	Wine, Wine (dry)	4.57	-	-	361.0796	4
Compound_13751	O-glycosyl compound	Total FB (dry), Wine, Wine (dry)	3.81	-	-	353.11	4
Compound_13753	Unknown	Wine, Wine (dry)	2.97	-	-	370.1373	4
Compound_14045	Unknown	Wine (dry)	3.67	377.0289	-	-	4
Compound_14065	Unknown	Wine, Wine (dry)	1.73	-	-	376.1217	4
Compound_14148	Unknown	Wine, Wine (dry)	3.72	-	-	396.0329	4
Compound_14466	Unknown	Wine, Wine (dry)	3.69	385.0083	-	-	4
Compound_14671	Unknown	Wine, Wine (dry)	2.99	389.1073	-	-	4
Compound_15147	Unknown	Total FB (dry)	2.05	-	-	417.1472	4
Compound_15246	Unknown	Wine, Wine (dry)	2.8	-	-	384.1258	4

Table S5. Features prioritized for identification from LC-MS positive mode (13 plasma, 89 urine)

Internal identifier	Identification	Food	RT (min)	m/z	Adducts	Measured neutral mass (Da)	ID level
Compound_15247	Unknown	Wine, Wine (dry)	1.98	402.1597	-	-	4
Compound_15433	Unknown	Wine, Wine (dry)	5.46	-	-	388.0997	4
Compound_15479	Unknown	Wine, Wine (dry)	2.81	407.1149	-	-	4
Compound_15481	Unknown	Wine, Wine (dry)	6.21	407.1329	-	-	4
Compound_15487	Unknown	Total FB	6	407.1386	-	-	4
Compound_16053	Unknown	Total FB (dry)	2.07	-	-	397.1574	4
Compound_16244	Unknown	Wine, Wine (dry)	6.21	424.1594	-	-	4
Compound_16323	Unknown	Wine, Wine (dry)	2.83	426.1595	-	-	4
Compound_17214	Unknown	Wine, Wine (dry)	1.98	445.1652	-	-	4
Compound_17215	Unknown	Wine, Wine (dry)	2.8	445.1652	-	-	4
Compound_17585	Unknown	Beer, Beer (dry)	3.77	-	-	435.163	4
Compound_17613	Unknown	Wine, Wine (dry)	2.69	454.1543	-	-	4
Compound_18170	Unknown	Wine, Wine (dry)	3.88	467.1175	-	-	4
Compound_19417	Unknown	Beer, Beer (dry)	6.84	-	-	482.1598	4
Compound_19773	Unknown	Wine, Wine (dry)	3.27	510.1804	-	-	4
Compound_19888	Unknown	Beer, Beer (dry)	7.43	514.2091	-	-	4
Compound_20504	Unknown	Beer, Beer (dry)	5.81	-	-	266.618	4
Compound_20505	Unknown	Beer, Beer (dry), Total FB (dry)	5.62	-	-	266.6181	4
Compound_20923	Unknown	Beer, Beer (dry)	6.35	-	-	273.6259	4
Compound_21904	Unknown	Wine, Wine (dry)	5.6	584.1963	-	-	4

FB; total fermented beverages; FCG, fermented cereals/grains; FD, fermented dairy; ID, identification; NA, not applicable; RT, retention time.

[a] Matched on retention time and mass; information on fragmentation not available. For hydroxy(iso)butyric acid, the internal standard used for identification was the sodium salt of (S)-3-hydroxyisobutyric acid; thus, a Level 2 identification was assigned instead of Level 1.

[b] Matched on retention time, mass, and fragmentation pattern. See Figure S4 for the obtained MSMS fragmentation pattern.

Table S6. Spearman's correlations between identified metabolites and self-reported fermented food intakes

Identification	Fermented foods and groups	Platform	Biosample	Spearman's correlations between metabolites and self-reported fermented food intakes														
				FFQ±14d			FFQ±30d			FFQ±90d			FFQ±180d			All FFQ		
				r	*p*-value	FDR adjusted *p*-value	r	*p*-value	FDR adjusted *p*-value	r	*p*-value	FDR adjusted *p*-value	r	*p*-value	FDR adjusted *p*-value	r	*p*-value	FDR adjusted *p*-value
Erythritol	Wine (dry)	GC-MS	Plasma	0.251	***	**	0.220	***	**	0.191	***	**	0.176	***	**	0.181	***	***
	Wine	GC-MS	Plasma	0.254	***	**	0.221	***	**	0.192	***	**	0.174	***	**	0.180	***	***
Quinate	Coffee (dry)	GC-MS	Plasma	0.441	***	***	0.443	***	***	0.424	***	***	0.409	***	***	0.401	***	***
	Coffee	GC-MS	Plasma	0.441	***	***	0.443	***	***	0.424	***	***	0.409	***	***	0.401	***	***
	Total FB (dry)	GC-MS	Plasma	0.288	***	***	0.271	***	***	0.227	***	***	0.214	***	***	0.207	***	***
	Total FB	GC-MS	Plasma	0.446	***	***	0.444	***	***	0.401	***	***	0.392	***	***	0.381	***	***
L-Phenylalanine	Total FB	GC-MS	Plasma	0.156	*	*	0.149	*	*	0.133	*	*	0.112	*	*	0.109	*	*
2-Hydroxybutyric acid	Total FB (dry)	GC-MS	Plasma	0.384	***	***	0.317	***	***	0.290	***	***	0.219	***	***	0.229	***	***
L-Cysteine	Beer (dry)	GC-MS	Plasma	0.210	**	**	0.172	**	*	0.173	**	**	0.144	**	**	0.146	**	**
	Beer	GC-MS	Plasma	0.210	**	**	0.172	**	*	0.173	**	**	0.144	**	**	0.146	**	**
	Total FB (dry)	GC-MS	Plasma	0.303	***	***	0.226	***	*	0.226	***	***	0.186	***	***	0.173	***	***
Dodecanoic acid [a]	White bread (dry)	GC-MS	Plasma	-0.128	*	0.438	-0.108	0.077	0.488	-0.085	0.118	0.745	-0.023	0.597	0.654	-0.027	0.533	0.699
Isoleucine	Total FB	GC-MS	Plasma	0.200	**	**	0.190	**	**	0.174	**	**	0.134	**	**	0.139	**	**
Xylitol	Total FB (dry)	GC-MS	Plasma	0.394	***	***	0.317	***	***	0.317	***	***	0.230	***	***	0.230	***	***
	Wine (dry)	GC-MS	Plasma	0.275	***	***	0.222	***	**	0.206	***	**	0.137	**	**	0.135	**	**
	Wine	GC-MS	Plasma	0.275	***	***	0.221	***	**	0.204	***	**	0.136	**	**	0.135	**	**
Glucuronic acid [a]	Wine (dry)	GC-MS	Urine	-0.163	*	*	-0.198	**	**	-0.235	***	***	-0.261	***	***	-0.268	***	***
m-Cresol [a]	Total FB	GC-MS	Urine	-0.185	**	**	-0.185	**	**	-0.175	**	**	-0.130	**	**	-0.133	**	**
	Total FB (dry)	GC-MS	Urine	-0.215	**	**	-0.176	**	**	-0.173	**	**	-0.120	**	**	-0.129	**	**
4-Hydroxybenzeneacetic acid	Total FB	GC-MS	Urine	0.254	***	***	0.279	***	***	0.264	***	***	0.215	***	***	0.219	***	***
	Total FB (dry)	GC-MS	Urine	0.334	***	***	0.330	***	***	0.323	***	***	0.249	***	***	0.256	***	***
	Wine	GC-MS	Urine	0.271	***	***	0.251	***	***	0.228	***	***	0.190	***	***	0.197	***	***
	Wine (dry)	GC-MS	Urine	0.266	***	***	0.246	***	***	0.225	***	***	0.189	***	***	0.196	***	***
	Beer	GC-MS	Urine	0.185	**	*	0.170	**	*	0.184	**	**	0.126	**	*	0.128	**	*
	Beer (dry)	GC-MS	Urine	0.185	**	*	0.170	**	*	0.184	**	**	0.126	**	*	0.128	**	*
3-Hydroxyhippuric acid	Total FB	GC-MS	Urine	0.214	**	**	0.267	***	***	0.299	***	***	0.336	***	***	0.333	***	***
Tartaric acid	Total FB (dry)	GC-MS	Urine	0.257	***	***	0.251	***	***	0.275	***	***	0.260	***	***	0.280	***	***
	Wine	GC-MS	Urine	0.388	***	***	0.353	***	***	0.384	***	***	0.398	***	***	0.417	***	***
	Wine (dry)	GC-MS	Urine	0.387	***	***	0.352	***	***	0.385	***	***	0.396	***	***	0.416	***	***
D-Gluconate	Wine	GC-MS	Urine	0.186	**	*	0.165	**	*	0.111	*	0.056	0.105	*	*	0.109	*	*
	Wine (dry)	GC-MS	Urine	0.185	**	*	0.164	**	*	0.111	*	0.057	0.106	*	*	0.110	**	*

Table S6. Spearman's correlations between identified metabolites and self-reported fermented food intakes

Identification	Fermented foods and groups	Platform	Biosample	Spearman's correlations between metabolites and self-reported fermented food intakes														
				FFQ±14d			FFQ±30d			FFQ±90d			FFQ±180d			All FFQ		
				r	*p*-value	FDR adjusted *p*-value	r	*p*-value	FDR adjusted *p*-value	r	*p*-value	FDR adjusted *p*-value	r	*p*-value	FDR adjusted *p*-value	r	*p*-value	FDR adjusted *p*-value
Catechol	Total FB	GC-MS	Urine	0.322	***	***	0.345	***	***	0.342	***	***	0.294	***	***	0.285	***	***
	Coffee	GC-MS	Urine	0.327	***	***	0.350	***	***	0.329	***	***	0.293	***	***	0.287	***	***
	Coffee (dry)	GC-MS	Urine	0.327	***	***	0.350	***	***	0.329	***	***	0.293	***	***	0.287	***	***
Guaiacol	Total FB	GC-MS	Urine	0.234	***	**	0.227	***	***	0.229	***	***	0.217	***	***	0.222	***	***
Glyceryl-glycoside TMS ether	Total FCG	GC-MS	Urine	0.365	***	***	0.367	***	***	0.311	***	***	0.291	***	***	0.289	***	***
	Total FCG (dry)	GC-MS	Urine	0.365	***	***	0.366	***	***	0.311	***	***	0.296	***	***	0.295	***	***
Citramalate	Wine	GC-MS	Urine	0.249	***	**	0.215	***	**	0.259	***	***	0.242	***	***	0.247	***	***
	Wine (dry)	GC-MS	Urine	0.247	***	**	0.214	***	**	0.258	***	***	0.244	***	***	0.248	***	***
Niacin (Nicotinate/Vitamin B3)	Total FB	GC-MS	Urine	0.421	***	***	0.401	***	***	0.341	***	***	0.282	***	***	0.275	***	***
	Total FB (dry)	GC-MS	Urine	0.244	***	***	0.256	***	***	0.232	***	***	0.172	***	***	0.176	***	***
	Coffee	GC-MS	Urine	0.462	***	***	0.425	***	***	0.356	***	***	0.306	***	***	0.301	***	***
	Coffee (dry)	GC-MS	Urine	0.462	***	***	0.425	***	***	0.356	***	***	0.306	***	***	0.301	***	***
D-Psicose [a]	Total FB	GC-MS	Urine	-0.368	***	***	-0.382	***	***	-0.341	***	***	-0.307	***	***	-0.312	***	***
	Total FB (dry)	GC-MS	Urine	-0.380	***	***	-0.387	***	***	-0.369	***	***	-0.324	***	***	-0.327	***	***
	Coffee	GC-MS	Urine	-0.227	***	**	-0.258	***	***	-0.232	***	***	-0.208	***	***	-0.217	***	***
	Coffee (dry)	GC-MS	Urine	-0.227	***	**	-0.258	***	***	-0.232	***	***	-0.208	***	***	-0.217	***	***
	Beer	GC-MS	Urine	-0.292	***	***	-0.275	***	***	-0.269	***	***	-0.223	***	***	-0.214	***	***
	Beer (dry)	GC-MS	Urine	-0.292	***	***	-0.275	***	***	-0.269	***	***	-0.222	***	***	-0.214	***	***
3,4-Dihydroxyhydrocinnamic acid	Total FB	GC-MS	Urine	0.210	**	**	0.206	**	**	0.159	**	**	0.152	***	**	0.167	***	***
D-Fucitol [a]	Coffee	GC-MS	Urine	-0.182	**	*	-0.146	*	*	-0.116	*	*	-0.105	*	*	-0.091	*	*
	Coffee (dry)	GC-MS	Urine	-0.182	**	*	-0.146	*	*	-0.116	*	*	-0.105	*	*	-0.091	*	*
Ethyl α-D-glucopyranoside	Total FB	GC-MS	Urine	0.230	***	**	0.199	**	**	0.241	***	***	0.233	***	***	0.252	***	***
	Total FB (dry)	GC-MS	Urine	0.395	***	***	0.389	***	***	0.412	***	***	0.388	***	***	0.394	***	***
	Wine	GC-MS	Urine	0.203	**	**	0.224	***	**	0.273	***	***	0.274	***	***	0.287	***	***
	Wine (dry)	GC-MS	Urine	0.202	**	**	0.223	***	**	0.274	***	***	0.277	***	***	0.290	***	***
	Beer	GC-MS	Urine	0.271	***	***	0.258	***	***	0.253	***	***	0.232	***	***	0.229	***	***
	Beer (dry)	GC-MS	Urine	0.271	***	***	0.258	***	***	0.253	***	***	0.232	***	***	0.229	***	***
Furoylglycine	Total FB	GC-MS	Urine	0.349	***	***	0.351	***	***	0.365	***	***	0.375	***	***	0.378	***	***
	Coffee	GC-MS	Urine	0.421	***	***	0.411	***	***	0.401	***	***	0.423	***	***	0.429	***	***
	Coffee (dry)	GC-MS	Urine	0.421	***	***	0.411	***	***	0.401	***	***	0.423	***	***	0.429	***	***
2-Keto-l-gluconic acid [a]	Total FB	GC-MS	Urine	-0.281	***	***	-0.267	***	***	-0.263	***	***	-0.254	***	***	-0.238	***	***

Table S6. Spearman's correlations between identified metabolites and self-reported fermented food intakes

Identification	Fermented foods and groups	Platform	Biosample	Spearman's correlations between metabolites and self-reported fermented food intakes: FFQ±14d			FFQ±30d			FFQ±90d			FFQ±180d			All FFQ		
				r	*p*-value	FDR adjusted *p*-value	r	*p*-value	FDR adjusted *p*-value	r	*p*-value	FDR adjusted *p*-value	r	*p*-value	FDR adjusted *p*-value	r	*p*-value	FDR adjusted *p*-value
	Coffee	GC-MS	Urine	-0.316	***	***	-0.301	***	***	-0.292	***	***	-0.263	***	***	-0.254	***	***
	Coffee (dry)	GC-MS	Urine	-0.316	***	***	-0.301	***	***	-0.292	***	***	-0.263	***	***	-0.254	***	***
Arabinofuranose	Total FB (dry)	GC-MS	Urine	0.257	***	***	0.269	***	***	0.272	***	***	0.257	***	***	0.260	***	***
	Wine	GC-MS	Urine	0.400	***	***	0.410	***	***	0.403	***	***	0.398	***	***	0.400	***	***
	Wine (dry)	GC-MS	Urine	0.397	***	***	0.406	***	***	0.397	***	***	0.394	***	***	0.397	***	***
D-Lactose	Total FB	GC-MS	Urine	0.226	***	**	0.233	***	***	0.222	***	***	0.188	***	***	0.200	***	***
	Total FB (dry)	GC-MS	Urine	0.246	***	***	0.262	***	***	0.263	***	***	0.221	***	***	0.232	***	***
	Beer	GC-MS	Urine	0.203	**	**	0.191	**	*	0.182	**	**	0.087	*	0.109	0.083	0.051	0.107
	Beer (dry)	GC-MS	Urine	0.203	**	**	0.191	**	*	0.181	**	**	0.087	*	0.108	0.083	0.051	0.107
Glycine [a]	Total FB	GC-MS	Urine	-0.199	**	**	-0.188	**	**	-0.192	***	**	-0.167	***	***	-0.161	***	***
3-deoxy-D-ribo-hexonic acid gamma-lactone	Total FB	GC-MS	Urine	0.378	***	***	0.386	***	***	0.394	***	***	0.363	***	***	0.356	***	***
	Total FB (dry)	GC-MS	Urine	0.211	**	**	0.229	***	***	0.266	***	***	0.200	***	***	0.205	***	***
	Coffee	GC-MS	Urine	0.394	***	***	0.395	***	***	0.390	***	***	0.387	***	***	0.380	***	***
	Coffee (dry)	GC-MS	Urine	0.394	***	***	0.395	***	***	0.390	***	***	0.387	***	***	0.380	***	***
2,3-Dihydroxypropyl phosphoric acid	Total FB	GC-MS	Urine	0.245	***	***	0.239	***	***	0.237	***	***	0.162	***	***	0.171	***	***
	Total FB (dry)	GC-MS	Urine	0.190	**	**	0.175	**	**	0.203	***	***	0.123	**	**	0.142	**	**
	Beer	GC-MS	Urine	0.210	**	**	0.176	**	*	0.220	***	***	0.139	**	**	0.136	**	**
	Beer (dry)	GC-MS	Urine	0.210	**	**	0.176	**	*	0.220	***	***	0.139	**	**	0.136	**	**
2,3-Dihydroxybutanoic acid	Total FB	GC-MS	Urine	0.241	***	***	0.202	**	**	0.234	***	***	0.142	**	**	0.164	***	***
	Total FB (dry)	GC-MS	Urine	0.261	***	***	0.218	***	**	0.279	***	***	0.206	***	***	0.232	***	***
	Beer	GC-MS	Urine	0.170	**	*	0.176	**	*	0.264	***	***	0.209	***	***	0.214	***	***
	Beer (dry)	GC-MS	Urine	0.170	**	*	0.176	**	*	0.264	***	***	0.209	***	***	0.214	***	***
Erythritol	Total FB (dry)	GC-MS	Urine	0.139	*	*	0.134	*	*	0.201	***	***	0.196	***	***	0.213	***	***
	Wine	GC-MS	Urine	0.315	***	***	0.285	***	***	0.314	***	***	0.312	***	***	0.327	***	***
	Wine (dry)	GC-MS	Urine	0.307	***	***	0.278	***	***	0.309	***	***	0.309	***	***	0.324	***	***
Trigonelline	Coffee	LC-MS	Plasma	0.308	***	***	0.315	***	***	0.303	***	***	0.343	***	***	0.352	***	***
	Coffee (dry)	LC-MS	Plasma	0.308	***	***	0.315	***	***	0.303	***	***	0.343	***	***	0.352	***	***
Glutamic acid	Coffee	LC-MS	Plasma	0.203	**	**	0.190	**	**	0.159	**	**	0.140	**	**	0.145	**	**
	Coffee (dry)	LC-MS	Plasma	0.203	**	**	0.190	**	**	0.159	**	**	0.140	**	**	0.145	**	**
Hydroxy(*iso*)butyric acid	Wine	LC-MS	Plasma	0.357	***	***	0.356	***	***	0.326	***	***	0.263	***	***	0.265	***	***
	Wine (dry)	LC-MS	Plasma	0.360	***	***	0.358	***	***	0.327	***	***	0.263	***	***	0.266	***	***

Table S6. Spearman's correlations between identified metabolites and self-reported fermented food intakes																			
**Identification**	**Fermented foods and groups**	**Platform**	**Biosample**	**Spearman's correlations between metabolites and self-reported fermented food intakes**															
				**FFQ±14d**			**FFQ±30d**			**FFQ±90d**			**FFQ±180d**			**All FFQ**		
				**r**	***p*-value**	**FDR adjusted *p*-value**	**r**	***p*-value**	**FDR adjusted *p*-value**	**r**	***p*-value**	**FDR adjusted *p*-value**	**r**	***p*-value**	**FDR adjusted *p*-value**	**r**	***p*-value**	**FDR adjusted *p*-value**
Cinnamoylglycine	Wine	LC-MS	Urine	0.350	***	***	0.370	***	***	0.381	***	***	0.392	***	***	0.384	***	***
	Wine (dry)	LC-MS	Urine	0.350	***	***	0.369	***	***	0.380	***	***	0.392	***	***	0.383	***	***
Methyluric acid	Coffee	LC-MS	Urine	0.490	***	***	0.490	***	***	0.504	***	***	0.491	***	***	0.493	***	***
	Coffee (dry)	LC-MS	Urine	0.490	***	***	0.490	***	***	0.504	***	***	0.491	***	***	0.493	***	***
Dimethyluric acid	Coffee	LC-MS	Urine	0.493	***	***	0.499	***	***	0.518	***	***	0.499	***	***	0.508	***	***
	Coffee (dry)	LC-MS	Urine	0.493	***	***	0.499	***	***	0.518	***	***	0.499	***	***	0.508	***	***

FFQ, food frequency questionnaire; GC-MS, gas chromatography mass spectrometry; LC-MS, liquid chromatography mass spectrometry. *, p<0.05; **, p<0.01; ***, p<0.001. The strength of the correlation coefficients are visualized along a colour gradient of red (positive correlations) to blue (negative correlations).

[a] Based on the Spearman's correlations this metabolite is negatively associated with the fermented foods and food groups indicated, but discriminant based on statistical significance in other tests (*e.g.*, PLS-DA, Random Forest).

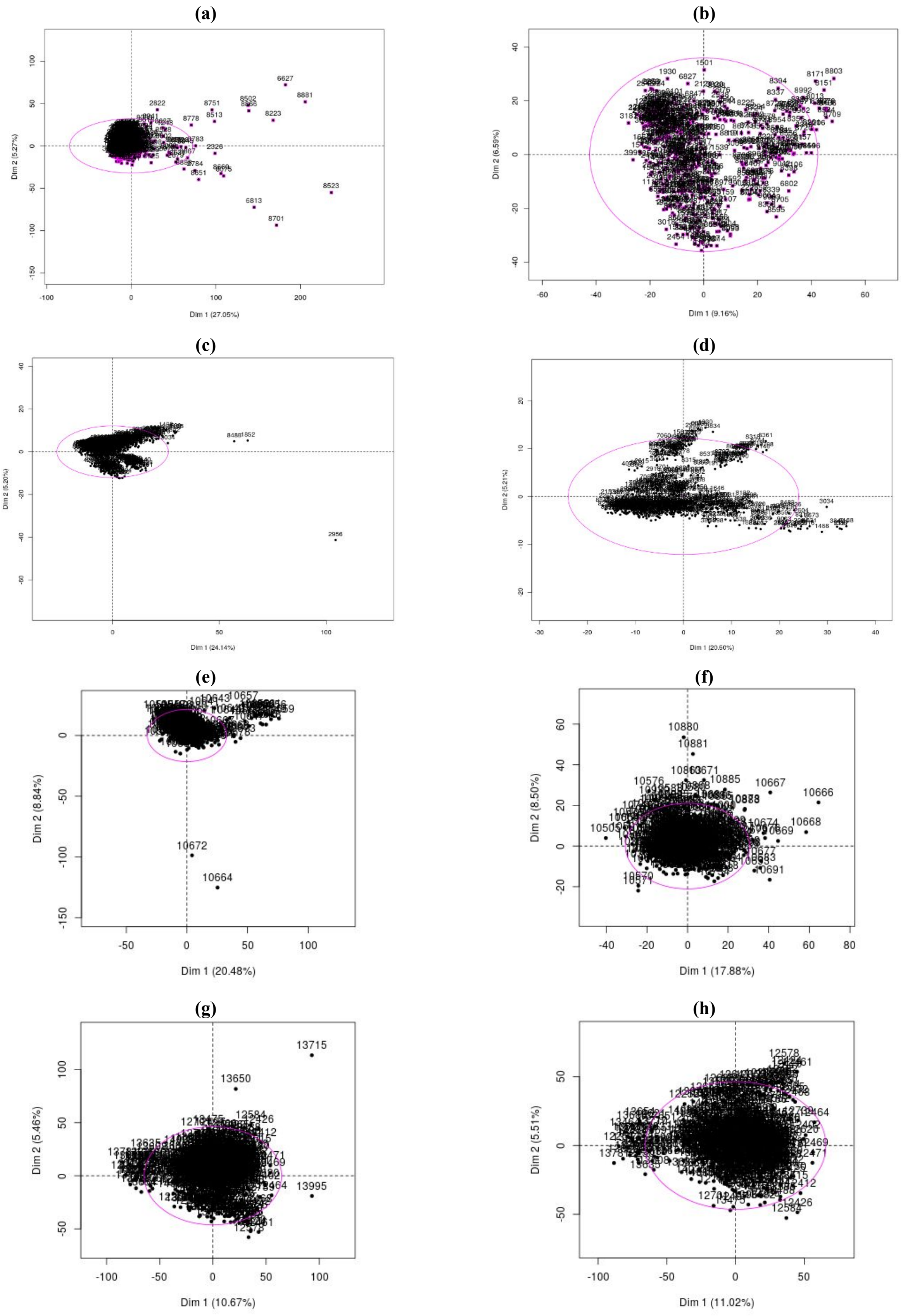

Figure S1. Principal Components Analysis (PCAs) of individual participant samples of the metabolomics subcohort prior to and after removal of outliers. Samples analysed by GC-MS: (a) plasma samples prior to outlier removal and (b) after outlier removal; (c) urine samples prior to outlier removal and (d) after outlier removal. Samples analysed by LC-MS: (e) plasma samples prior to outlier removal and (f) after outlier removal; (g) urine samples prior to outlier removal and (h) after outlier removal.

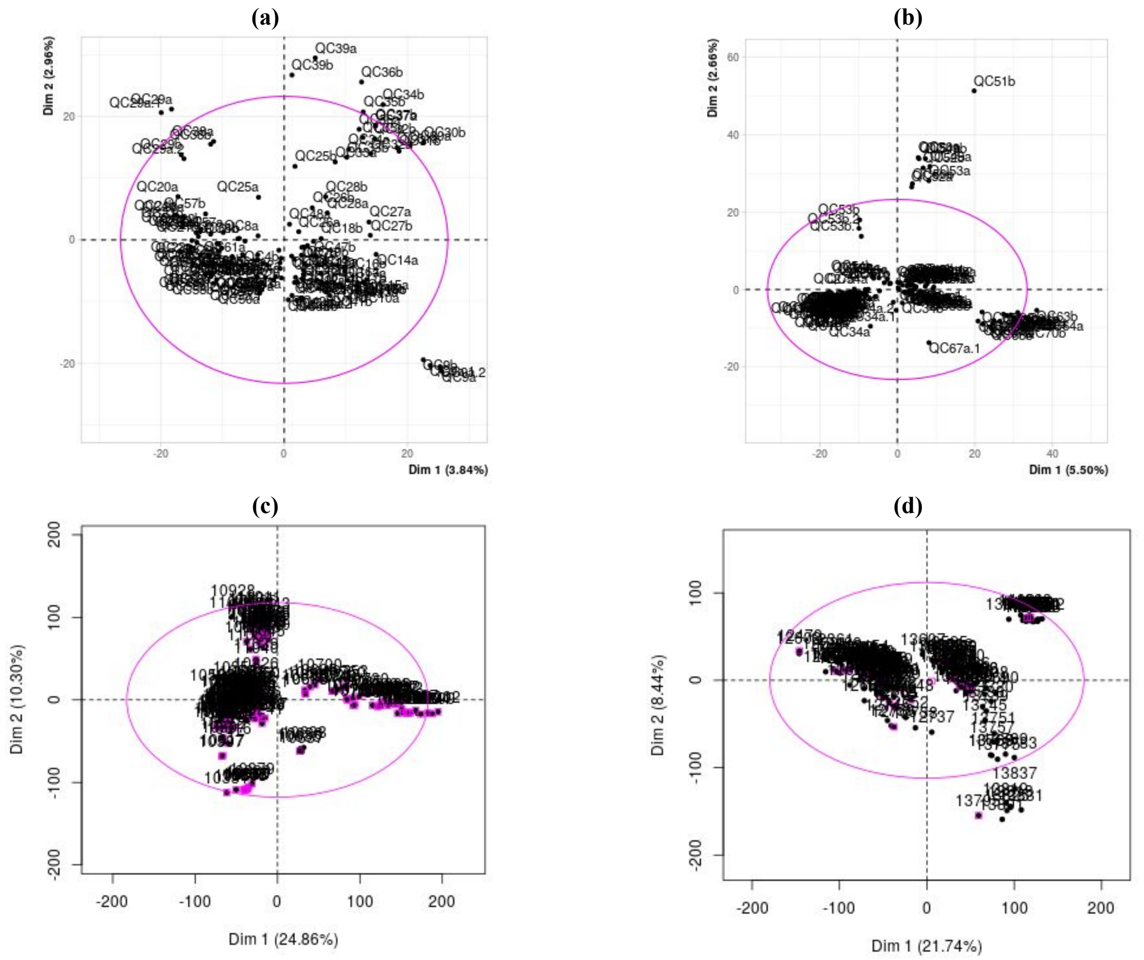

Figure S2. Principal Components Analysis (PCAs) of quality control samples analysed by GC-MS: (a) plasma and (b) urine; LC-MS (c) plasma and (d) urine.

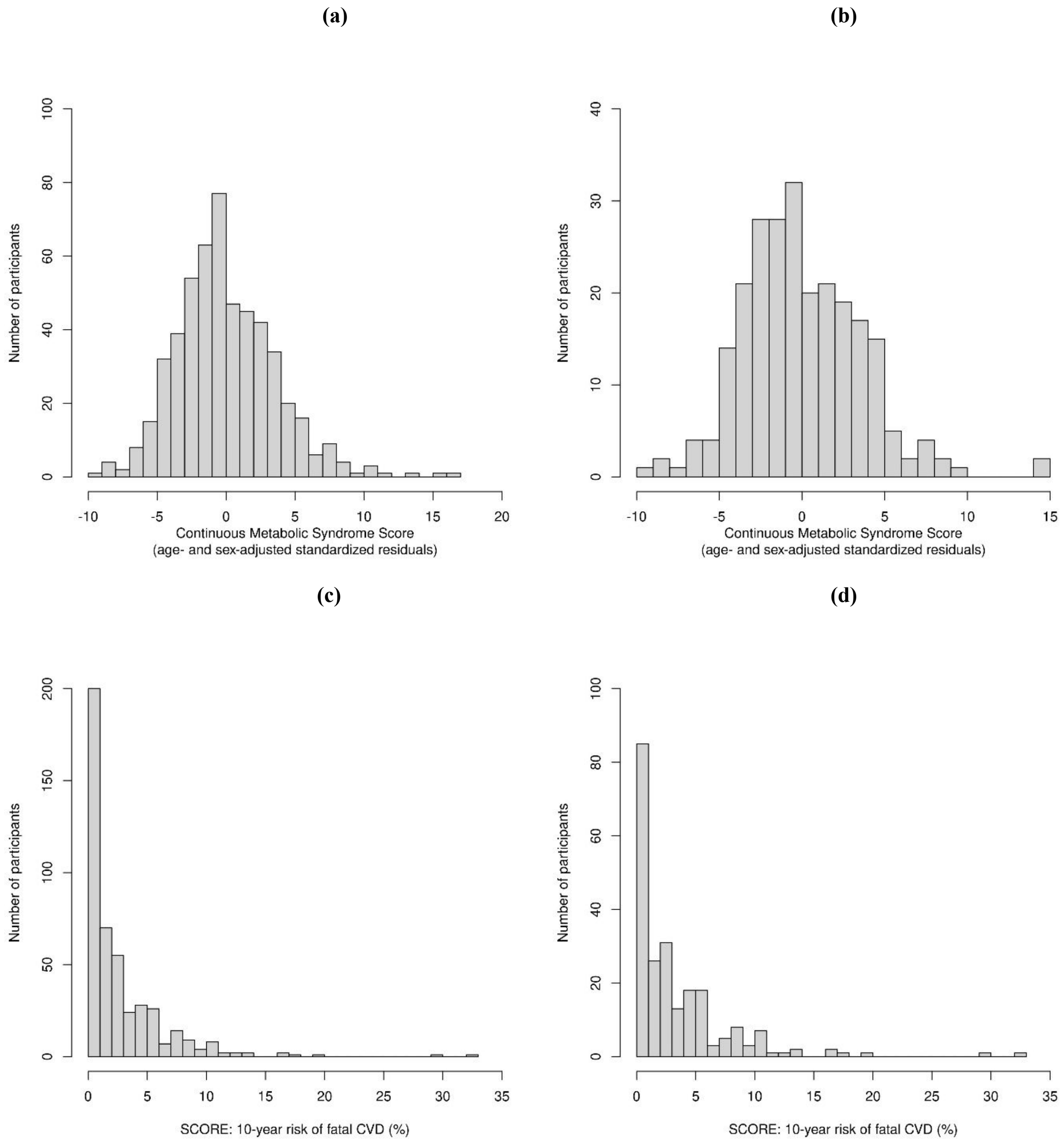

Figure S3. Distribution of (a) continuous metabolic syndrome in (a) the metabolomics subcohort (*n* = 531) and (b) the identification subcohort (*n* = 246). Distribution of (c) SCORE in the metabolomics subcohort (*n* = 531) and (b) the identification subcohort (*n* = 246).

(a)

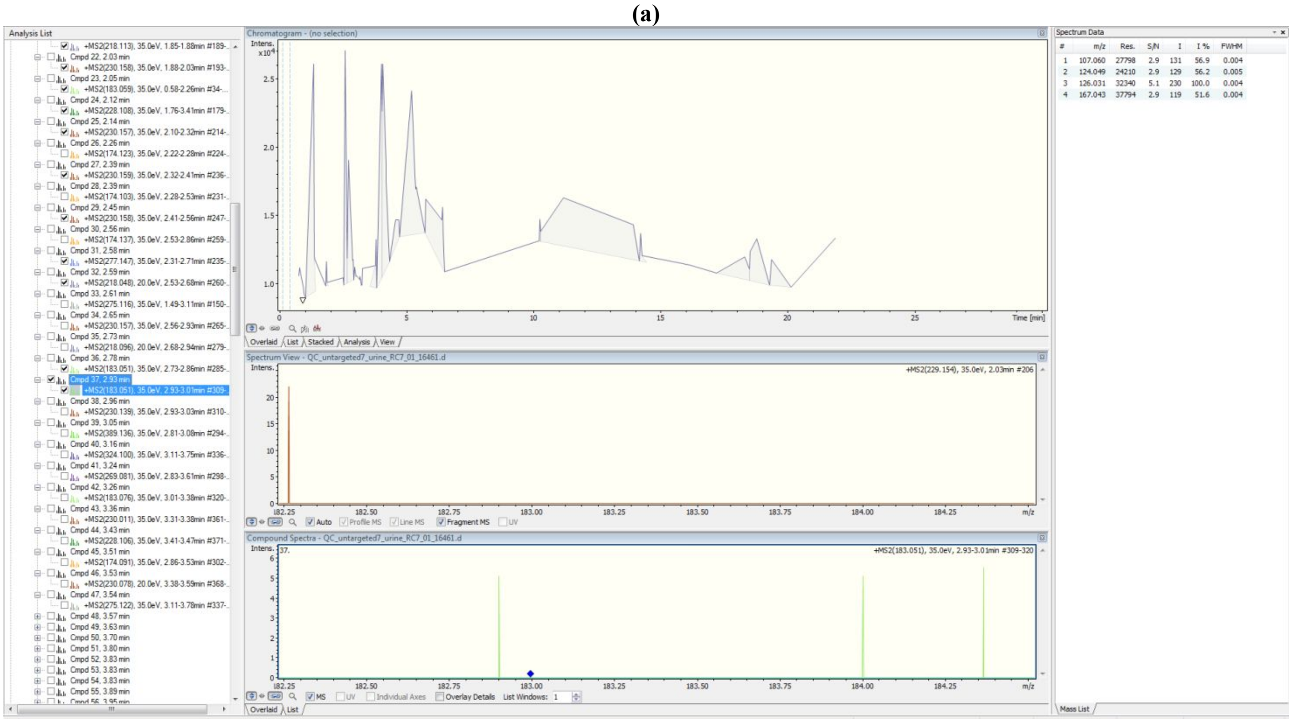

(b)

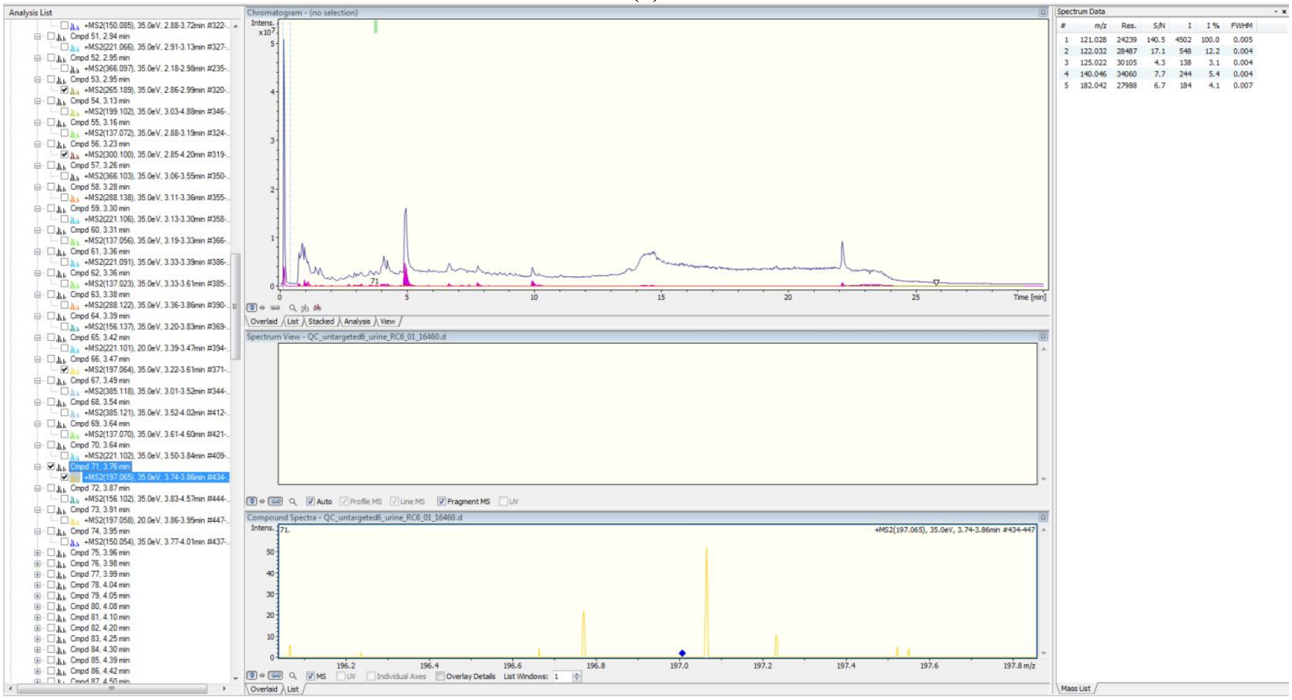

Figure S4. LC-MS fragmentation data for (a) Compound_3885 identified as methyluric acid (based on a suggested match to 1-methyluric acid MSMS spectral data on Metlin and MSMS of internal standard) and (b) Compound_4443 identified as dimethyluric acid (based on suggested matches to 1,3- and 3,7-dimethyluric acid MSMS spectral data on Metlin and MSMS of internal standard).
